# Protecting hidden treasures: Indigenous lands safeguard 50% of areas with the highest potential for angiosperm discoveries in Brazil—patterns and conservation priorities

**DOI:** 10.1371/journal.pone.0326507

**Published:** 2025-07-09

**Authors:** Janaína Gomes-da-Silva, Eimear Nic Lughadha, Rafaela Campostrini Forzza

**Affiliations:** 1 Jardim Botânico do Rio de Janeiro, Rua Pacheco Leão, Rio de Janeiro, Brazil; 2 Science Directorate, Royal Botanic Gardens, Kew, Richmond, United Kingdom; 3 Instituto Chico Mendes de Conservação da Biodiversidade, Parque Nacional do Descobrimento, Bahia, Brazil; University of Mississippi, BRAZIL

## Abstract

Brilliantly adapted from aphorist and geneticist Dobzhansky’s phrase, “Nothing in biology makes sense except in the light of taxonomy” conveys the fact that various scientific fields depend on correctly identified and accurately described species. In this sense, estimating the number of extant species is one of the fundamental issues and has direct implications for biodiversity conservation. Worldwide, approximately 370,000 angiosperm species are known to science; however, studies indicate that another ~100,000 or as many as 315,000 are yet to be described for science. Brazil is recognized for its megabiodiversity and currently recognizes 32,900 native species of angiosperms. What would be the impact on conservation priorities if all undescribed species were known and the catalog was complete? To explore this, we analyzed datasets of taxonomic information available for Brazilian angiosperms published between 1753 and 2020 to understand patterns of species discovery and identify which areas in which Brazilian phytogeographic domains harbor the largest number of species unknown to science. The likely number of species remaining to be described was extrapolated using predictive models and incorporating taxonomic effort over time. We estimated that the catalog of Brazilian angiosperms is at least 19–23% incomplete, with the proportion incomplete ranging from 4% to 39% across phytogeographic domains, and 7343–9595 species still awaiting description. Despite differences between models, overall trends consistently indicate the Amazonia and Caatinga regions offer greatest potential for new species descriptions. Our analysis revealed that human population density explains more variation in rates of angiosperm description over centuries than taxonomic effort, but taxonomic effort is a better predictor of recent description rates. Worryingly, 80% of areas predicted to be richest in undescribed angiosperm species do not overlap with protected areas (PAs), but 50% are within Indigenous lands. These findings highlight the urgent need to expand collection efforts and PAs coverage, particularly in the Caatinga, which has low levels of protection, and in Amazonia, where collaboration with indigenous communities is crucial for biodiversity conservation. Without direct action, many undescribed species and their undocumented traits and potential may be lost.

## Introduction

As brilliantly adapted by Rouhan & Gaudeul [1] from the remarkable phrase of the aphorist and geneticist Theodosius Dobzhansky, “Nothing in biology makes sense except in the light of taxonomy” [2]. It can be argued that the meticulous analysis and arrangement of species within a comprehensive framework forms the cornerstone of all biological inquiry, uniting the different areas of the biological sciences that depend on taxonomy, i.e., correctly identified and accurately described species [1,3–5]. The absence of a scientific name for a species makes it almost imperceptible to science, rendering it much less likely to be the focus of basic research or to be considered in conservation planning, and therefore more susceptible to extinction [6]. In this sense, estimating the number of living species on the planet as accurately as possible has been one of the enigmatic, fundamental, and most frequently asked questions in the biological sciences, with direct implications for biodiversity conservation [7,8,9,10,11].

Estimating the exact number of species on Earth is a daunting and controversial task. Current estimates, based on experts opinions, range from 3 million to over 100 million species [9,12]. Of these, only 1.2–1.8 million have been formally described and catalogued [9,13]. This means that the vast majority of species remain unnamed, undescribed, and unclassified, awaiting description [9,13,14].

Currently, around 325,000–370,000 extant species of angiosperms are known to science, although this number is controversial as estimates vary depending on biases arising from synonyms, taxonomic species delimitation or circumscription used, available data, and discrepancies stemming from changes in recent publications [6,15,16]. For example, four current vascular plant checklists provide identical information on just c. 60% of plant names [17]. Studies indicate that another ~ 300,000 [9] plants or ~100,000 angiosperm species [18] are still unknown to science and await description, henceforward we will use the term “undescribed” species. This number illustrates the limits to our taxonomic knowledge of the world flora despite 260 years of discovery and description of species since Linnaeus [1,19].

Brazil is the most floristically diverse country globally, with approximately 32,900 native angiosperms species, including 19,247 endemics [20,21], and 341 genera are unique to Brazil [22]. Rates of description of new angiosperm species have remained high in recent decades, and since the 1990s Brazil has been among the four countries with the highest number of annual descriptions [1,6,23,24]. However, a significant portion of the Brazilian flora remains unexplored [25, 26, 27], corroborating the probable incompleteness of the taxonomic catalog of this flora. This lack of knowledge leads to the existence of species going unnoticed and their unique attributes undocumented, leaving them at risk of being lost due to a lack of knowledge. Globally, most of the plant species described since 2020 and those yet to be described are likely to be recognized as threatened with extinction, once assessed [28]. Similarly, many of the endemic species of the Brazilian territory are threatened and may potentially be extinct before we can collect and describe them [6,9,29,30]. As a result, there is a critical need to inventory extant life on Earth, as well as to shift the paradigm that animal conservation is more important to fund than plant conservation [see more discussion in [31,32]. In this sense, identifying gaps in species knowledge is critical for identifying priority areas for new collection efforts [e.g., 33].

It is probably true that certain areas should receive priority in conservation efforts due to the potential increase in our understanding of species diversity within them and the extensive biodiversity and incomplete taxonomic catalog of flora. The difficulty, though, is in precisely delimiting these areas.

To estimate the total number of species including undescribed species, two questions must be answered. The first question seems simple from the layperson’s point of view but has proved practically impossible to answer with the consensus of all specialists. The question is “how many species have already been described in the studied region?” [1,8]. Based on twelve years of research, for Brazil, this question was recently answered by more than 900 taxonomists collectively titled “The Brazil Flora Group – BFG” [21,34]. The second question is more complex to answer because it requires estimation of the number of species as-yet-unknown to science, here termed “how many species remain undescribed?” [1,8]. Consequently, estimating the total number of species is enormously challenging [8].

To estimate the likely future description rates of new species, recent studies employ distinct methods of extrapolation of the number of species described over time or patterns of description date, the number of taxonomists active per year, taxonomic effort over time, while other studies use predictors, such as biological attributes, climatic conditions, rarity, geographic range, and other environmental and sociological attributes, at the species level [8,9,14,35,36].

The number of angiosperms yet to be described has previously been predicted using methods such as counting taxa on a global or continental scale [8], including all domains of life (e.g., algae, animals, fungi, plants, etc.), both on land and in the ocean [e.g., 9], and extrapolating the number of species to higher and lower taxonomic ranks [8,9]. However, no publication to date incorporates information from all the angiosperms recorded in the six Brazilian phytogeographic domains (i.e., Amazonia, Atlantic Forest, Caatinga, Cerrado, Pampa and Pantanal), integrating both old and newly collected data, with expert estimates of taxonomic effort and supported by empirical data allowing for a comprehensive view of species richness and of species description patterns.

Accordingly, our question is “What would be the impact on conservation priorities if all undescribed species were known and the catalog was complete or approaching an asymptote?”. Specifically, we seek to answer the following questions: (1) What were the patterns of angiosperm species description between 1753 and 2020? (2) Has there been a discernible change in taxonomic research efforts in recent decades, either an increase or decrease? (3) Are numbers of Brazilian angiosperms known to science approaching asymptotes? What factors contributed to botanical discoveries and consequent descriptions in Brazil? (4) Which phytogeographic domains have the greatest potential for the discovery of new species, and how does this potential vary across domains? (5) Which families have the greatest potential for the discovery of new species in the future? (6) Is there a spatial overlap between the areas with the highest future discovery potential and Brazil’s designated protected areas (PAs) and Indigenous lands?

## Materials and methods

### General procedures descriptive and predictive

For data description and predictive analyses, protocols for data review were developed by year using filter tools in Microsoft Excel v. 16.0 (Microsoft Office 2019 Proofing Tools).

Aiming to make the best use of the data, we conducted descriptive analyses by phytogeographical domain and number of species described over time (i.e., description dates), as well as by habit between 1753–2020. In addition, simple plots were produced for all families of angiosperms containing more than 500 species native to Brazil [34] to depict change in rates of species description over time.

### Species list = sampling taxa, data collection and data cleaning

The geographic scope comprises the national territory of Brazil, which extends from 5° to –34° Latitude; –34° to –73° Longitude and encompasses a total area of ~ 8.5 million km² [37]. Data on the description, distribution, and habit of ~32,900 native species belonging to 235 families were used, including all species of basal angiosperms, monocotyledons, and eudicotyledons known to occur in the six Brazilian phytogeographic domains, i.e., Amazonia, Caatinga, Cerrado, Atlantic Forest, Pampa, and Pantanal. Non–native species records were excluded from the analyses.

We assembled full datasets containing accepted species names from the International Plant Names Index [38], database; https://www.ipni.org/], an authoritative source of objective nomenclatural data that collates and indexes nomenclatural acts (including spelling, author(s), nomenclatural type(s), place, and year of description of taxa). Datasets of accepted species and their year of first description were assembled for all native Brazilian flowering plants. To delineate the IPNI subset, we utilized the accepted species list from the Flora and Funga of Brazil (2022). We applied a protocol to clean (both manually and automatically) the datasets and then insert missing data using the “filter” tool in Microsoft Excel v. 14.5 (Microsoft Office 2010 Proofing Tools), with the following steps:

(i) Data on genus and section names were excluded; only records at species and infraspecific rank remained in the database.
(ii) All data from IPNI were cross–checked with the list of angiosperms of Flora e Funga do Brasil 2022 [34] (http://floradobrasil.jbrj.gov.br).
(iii) Missing data were added manually. Data were checked to eliminate repeated names, i.e., duplicates with the same names at species level and the same protologue.
(iv) Each species name and infraspecific name in the dataset was assigned to a family following the APG IV [39] for high–level classification: e.g., the families Alliaceae in Amaryllidaceae; Asclepiadaceae in Apocynaceae; Avicenniaceae in Acanthaceae; Caesalpiniaceae, Leguminosae and Mimosaceae in Fabaceae; Buddlejaceae in Scrophulariaceae; Callitrichaceae in Plantaginaceae; Myrsinaceae in Primulaceae; and Sterculiaceae in Malvaceae.
(v) We obtained the year of description for each species name and infraspecific name from IPNI’s ‘publication_year_i’ field. In the absence of the information in this field, we completed the information manually with the information from IPNI’s ‘reference_t’ field. For the c. 10% of species where description year was not recorded in either field, we manually added the missing dates, obtained by consulting the original publication or Tropicos (https://www.tropicos.org/home).
(vi) The resulting list was uploaded in Plantminer (http://www.plantminer.com/) using Brazilian Flora – Flora R package [40] frontend to check habitat, phytogeographic domain, life form, and threat status for each taxon.
(vii) Per year and for each 10–year interval between 1753–2020, we calculated the number of species described, change in description rate over time, and species accumulation curve over time, for Brazilian angiosperms, separately for the six Brazilian phytogeographic domains and for the 16 families with more than 500 species, resulting in 23 datasets. These analyses were performed with two different data sets: (*i*) including all accepted names of species native to Brazil and their synonyms, designated as “all names,” and (*ii*) including only basionyms (i.e., excluding both homotypic and heterotypic synonyms, leaving just one (earliest) name to represent each currently accepted species). The basionym dataset was considered to offer the best solution to track progress towards completing the inventory of the Brazilian Flora through the addition of species previously unknown to science or not previously reported for Brazil. The all names dataset captures more of the breadth of activities in which taxonomists engage while documenting a flora, encompassing the full range of taxonomic effort including lumping and splitting species, transferring species from one genus to another, and other activities many of which result in purely nomenclatural changes that reflect our improved understanding of the flora and add to the number of names at species level without necessarily adding to the number of species recognized in Brazil.

### Attributes

To infer the probability of species description in the future and to explore the potential factors influencing patterns of species discovery over time in angiosperms, we utilized a set of attributes, as detailed below:

(i) **Year of species description**

For species discovery models, we used data between 1753–2020. The date 1753 was chosen as a starting point for the scientific names of plants from Linnaeus’ Species Plantarum. We only employed data until 2020 to avoid potential under-representation of names published in recent years due to delays between publication of species new to science and their inclusion in global databases, thus the year 2020 denotes the last year of data that we used to fit our models.

For the dataset entitled “all names” the data consist of the year of first publication for all scientific names associated with each native Brazilian angiosperm species. This includes all accepted names including infraspecific names, and their synonyms and basionyms.

For the dataset entitled “basionyms”, the data consist of the year of first publication of a scientific name at species level for each Brazilian angiosperm species, starting with Linnaeus (1753) and concluding in the year 2020. For this attribute, the event of interest is the date of first description of each species native to Brazil currently accepted as distinct. Since the names by which a species is known may have changed over time for a range of taxonomic and/or nomenclatural reasons, the date of first description of a species may precede the date of publication of its current accepted name by many years. Finding the date required for our analysis required consideration of all the synonyms associated with each accepted name, to find the basionyms. Importantly, consideration of the synonymy of each accepted name was not confined to BFG but involved consultation of other major databases. For species for which the basionym determination is discordant between databases, we adopted the following protocol to identify the correct basionyms:

For each name accepted in the Flora do Brasil, three databases were consulted: Flora e Funga do Brasil [34], Tropicos, and the World Checklist of Vascular Plants (WCVP) [https://www.gbif.org/dataset/f382f0ce–323a–4091–bb9f–add557f3a9a2]. In cases where the basionym indication from these three sources does not match, we applied majority rule to select the correct name, if two of the three sources agree, we used that name. This protocol minimized the inconsistency of basionym determination across the datasets.

(ii) **Taxonomic effort**

To estimate taxonomic effort the algorithm (details in Supporting Information, S1 Appendix) described by Joppa et al. [8] was used for the dataset of described species for Brazil between 1753–2020, for both all names and basionym datasets. In addition, to provide a more recent perspective on the drivers of taxonomic effort, we specifically analyzed data from the last four decades (1960–2020) to evaluate the relationship between taxonomic effort and human population density. According to the model, the greater the effort, i.e., the number of taxonomists involved in the description of species, the more species they will describe in each interval. In other words, the greater the number of taxonomists active in a family or region, the greater the probability that as–yet-undescribed species in that family or region may receive enough attention to be recognized as distinct and described by taxonomists. Conversely, a species may remain unknown if taxonomic activity is low.

(iii) **Human Population Density**

Previous studies have demonstrated a positive relationship between human density and the year of description of species [14,41]. Human occupancy data per km^2^, within–range human population density at the year in which the species was described as new to science (https://www.ibge.gov.br/estatisticas/sociais/populacao/9662–censo–demografico–2010.html?edicao=9754&t=series–historicas) was extracted from the Brazilian Agency of Geography and Statistics– IBGE. To calculate the influence on the description of species, we quantify the average human density within the currently known range of the species in the year of its description.

(iv) **Distribution data**

For our analyses, we gathered, evaluated, and, where necessary, modified distributional data for ~32,900 angiosperm species native to Brazil downloaded from the Global Biodiversity Information Facility (GBIF; < www.gbif.org>), and Reflora Virtual Herbarium (http://reflora.jbrj.gov.br/reflora/herbarioVirtual/), using collection records from collections, including 3.1 million voucher specimens (ALCB, ASE, BRBA, CEN, CEPEC, CESJ, CGMS, COR, CRI, DVPR, EAC, ECT, ESA, EVB, FIG, FLOR, FURB, HACAM, HBR, HCF, HDCF, HEPH, HRCB, HSTM, HTO, HUCO, HUCP, HUEFS, HUEM, HUEMG, HUENF, HUFU, HUNEB, HUNI, HUPG, HVASF, IAN, IBGE, ICN, LUSC, MAC, MBM, MBML,MG, MUFAL, PEL, PMSP, R, RB, REAL, RFA, UERJ, RFFP, RON, SJRP, SPF, UB, UFRN, UNIP,UNOP, UPCB, VIES, K, B, E, GH, MO, NY, P, S, US, W; acronyms follow Thiers, 2014,continuously updated). The GBIF and REFLORA databases were queried using only the native families of Brazil. Additionally, we used data of Gomes-da-Silva et al. [42]. Information for c. 4 million angiosperm specimen records were considered fit–for–use after the data cleaning (details in Supporting Information, S2 Appendix).

We conducted a completeness analysis, operating on the premise that areas with lower completeness of taxonomic inventories and low collection density, particularly in underexplored regions, hold the greatest potential for new species discoveries. To achieve this, we utilized databases of georeferenced records for Brazilian angiosperms and conducted a correlation and comparison of domain completeness with density of occurrence records through the following steps (Details of the analyses and completeness by polygon can be found in the Supporting Information, S1 Fig).

1. Mapping Areas with Low Completeness: We identified areas within each domain that exhibit low completeness and the broadest gaps by examining the number and distribution of the occurrence records. The distribution of each species was mapped using QGIS 3.28.5 (QGIS Geographic Information System, Open Source Geospatial Foundation Project, http://www.qgis.org/) at different spatial resolutions of 1/10° and ½°, as well as grid sizes of 1° x 1° and 2° x 2°.
  1. Density Analysis: We created a geohash multi-layer density map using all available occurrence records, including type specimens, in QGIS with the Density Analysis plugin (https://github.com/NationalSecurityAgency/qgis-densityanalysis-plugin). The records (data points) were spatially grouped into raster grids. For each polygon grid (156 km x 156 km, geohash resolution), the number of records was counted using the “Count Points in Polygon” algorithm, with an equal count (quantile) classification. The spatial density distribution was then refined and analyzed using Python, applying a 10-color scale to ensure accuracy in identifying underexplored regions.
  2. Overlaying with Density Analysis: We overlaid these low-completeness areas, or areas with fewer described species relative to their potential diversity, with the collection density map. Regions exhibiting both low collection density and low completeness are prime candidates for new collection expeditions, as they highlight underexplored areas with significant untapped biodiversity potential.
  3. Prioritization: We compared and integrated the completeness of the taxonomic inventory, identified gaps, and density analysis results to determine areas that coincide with domains or subregions requiring additional collection efforts. We prioritized areas with low collection density, insufficient taxonomic completeness, and unexplored regions with high potential for new species discovery. The final map, ranging from 0 (dark green, low priority) to 10 (white, high priority), highlights these priority areas, representing the greatest discovery opportunities.

### Taxonomic effort estimated by taxonomists

We contacted 979 taxonomy experts from *Flora do Brasil* (2020) via e–mail (details in Supporting Information, S4 appendix) of which ~ 510 responded to our survey, i.e., a response rate of 52.1%. The respondents were asked to identify whether the taxonomic effort (measured by the number of taxonomists) in their area of expertise has increased, decreased, or remained stable over the last four decades, and more specifically over the decade ending 2020. The results are compared with trends in the names dataset and discussed.

### Statistical analysis

To model the probability of species description and to predict the number of species remaining to be described in the future using both all names and basionyms datasets we fitted predictors and models that incorporate: (*i*) the year of description, and a model that incorporates taxonomic effort over time [8 more details in Supporting Information, S1 Appendix]; (*ii*) asymptotic regression models with a multimodel approach, using a variety of nonlinear models (Supporting Information, S1 Appendix).

In theory, as our understanding of the total species pool approaches the actual value, the cumulative count of newly described species should start to level off or approach an asymptotic pattern. Thus, to fit asymptotic regression models to predict the number of species, we used the four nonlinear models: Gompertz 3P, Gompertz 4P, Logistic 3P, and Weibull Growth (Supporting Information, S1 Appendix). As comparative studies have suggested that the results of parametric and non–parametric models to estimate species richness through the extrapolation of discovery record data may change substantially depending upon different attributes of the data [43], we used a multimodel approach and a model averaging approach to combine the results of different models. This procedure allowed us to weight the models based on their support of the data to achieve a more accurate statistical result than might be obtained through use of a single model. To evaluate the model performance of the approaches, we used the AIC (Akaike’s Information Criterion— lower AIC values indicate a better model), AIC Weight (Akaike weight, A model whose Akaike weight approaches 1 is unambiguously supported by the data), and BIC (Bayesian Information Criterion —lower BIC values indicate a better model) [44]. We calculated the uncertainty between different models and weighted their predictions based on how well they fit the data, using Akaike weight [44]. This multimodel averaging was used to compute the asymptotic number of species predicted. In addition, we used the multimodel weighted average unconditional standard errors to calculate 95% confidence intervals.

In summary, we used four different modeling approaches (i.e., Gompertz 3P, Gompertz 4P, Logistic 3P, and Weibull Growth) applied to each of the 46 datasets, being 23 datasets of “all names” and 23 datasets of “basionyms” (all angiosperms + 6 domains + 16 angiosperm families with > 500 species), with each dataset being divided into 10 year intervals. Analyses scripts available at Zenodo (https://zenodo.org/; https://doi.org/10.5281/zenodo.15202988).

The approaches that incorporate the year of description, and a model that incorporates taxonomic effort over time were applied to each of 14 datasets, i.e., all angiosperms + 6 domains, with seven including “all names” and seven only “basionyms”.

The models were fitted using R version 4.3.1 (R Core Team, 2023) R software, The R Foundation/ R Development, Core Team, https://www.rproject.org, JMP Statistical Discovery Software (SAS Institute Inc, 2023), and Stata (Corp. 2023. Stata Statistical Software: Release 18. College Station, TX: StataCorp LLC).

In addition, we used scatterplots and multiple regressions to assess the correlations between human population density and the number of described species, and between taxonomic effort and the number of described species using Excel and JMP Statistical Discovery Software (SAS Institute Inc, 2023).

To analyze the overall trend over time and observe how the accumulation of species descriptions correlates with human population density or the number of taxonomists, and to understand the general progress throughout the period, we used the cumulative number of species described per decade. However, to provide a more recent overview of the taxonomic effort, we evaluated the last four decades (1960–2020) using individual numbers per decade to highlight how the conditions of each decade influenced the description of new species, capturing fluctuations and potential periods of increased or decreased taxonomic activity.

Finally, the predicted number of species from the models was used to calculate the completeness of the taxonomic inventory as follows: after obtaining the “estimated total species richness, asymptote” for each phytogeographic domain using statistical models, the Completeness Index (S1 Appendix) was employed to measure to what extent the observed species richness represents the total estimated species richness in each area.

### Conservation of areas with highest number of species unknown to science

With the purpose of determining whether areas with the highest number of angiosperm species unknown to science are located within or near Ministry of Environment protected areas

(PAs, [45], indigenous lands (Terras Indígenas TI, https://www.gov.br/funai/pt–br/atuacao/terras–indigenas/geoprocessamento–e–mapas) or fire spots (focus points) based on INPE (Instituto Nacional de Pesquisas Espaciais) data (https://queimadas.dgi.inpe.br/queimadas/exportacaobdq/download?token=6581b2b8–a5eb–9a32–a687–d46f7dcc3cab), the maps of these areas were plotted against the priority areas identified in our study. The shapefiles of PAs [45], Indigenous lands, and focus points were plotted using QGIS. Lastly, the areas recovered in the analysis were superimposed to identify areas inside and outside PAs and fire focus points. In addition, cumulative historical deforestation up to 2022 was retrieved from the Amazon Deforestation Estimation Project (PRODES) to correlate with collection efforts up to 2020 in the region (details in Supporting Information, S7 Appendix).

**Fig 1:**
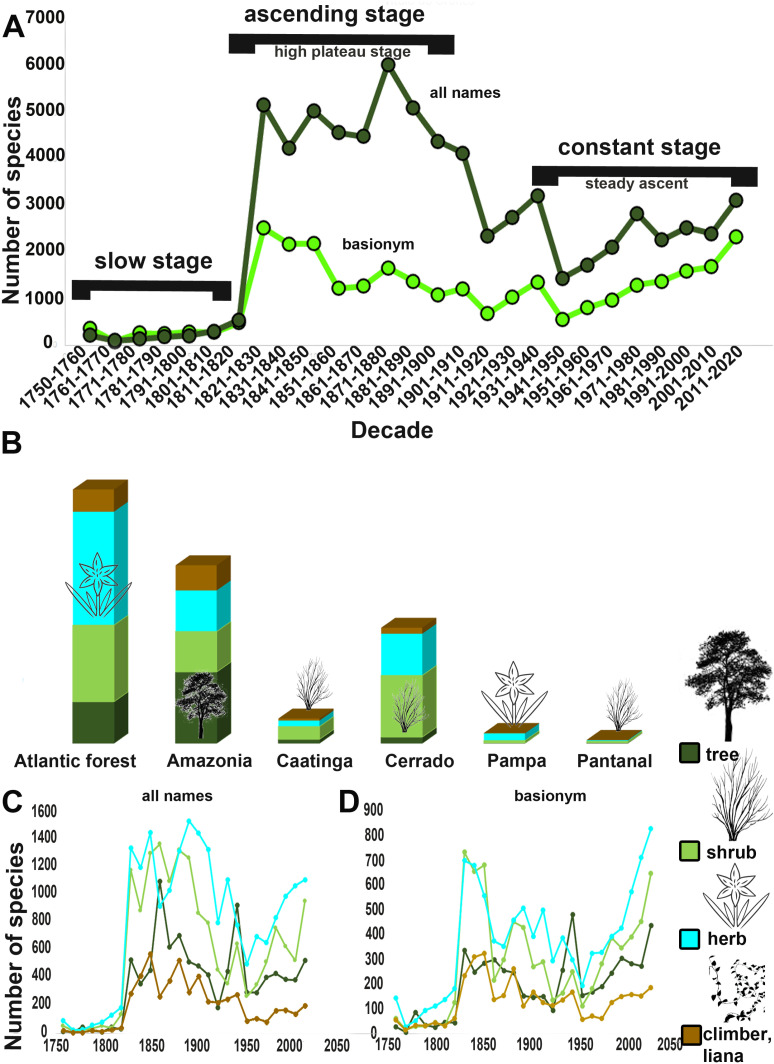
Taxonomic information available to Brazilian angiosperms published between 1753–2020. A. Number of angiosperm names described as new to science in each decade from 1753 to 2020, based on data from IPNI (International Plant Name Index, https://www.ipni.org/) and Flora e Funga do Brasil, 2023 A. Temporal description of angiosperm species in Brazil. B. Proportions of tree, shrub, herb, climber, and liana species for each phytogeographical domain. C. Number of angiosperm names published as new to science in each decade from 1753 to 2020, plotted by type of habit tree, shrub, herb, climber, and liana according to Flora e Funga do Brasil, (2022), per domain for dataset “all names”. **D.** Number of angiosperm names described as new to science in each decade from 1753 to 2020, plotted by type of habit tree, shrub, herb, climber, and liana according to Flora e Funga do Brasil, (2022), per habitat and per domain for dataset “basionym”.

**Fig 2:**
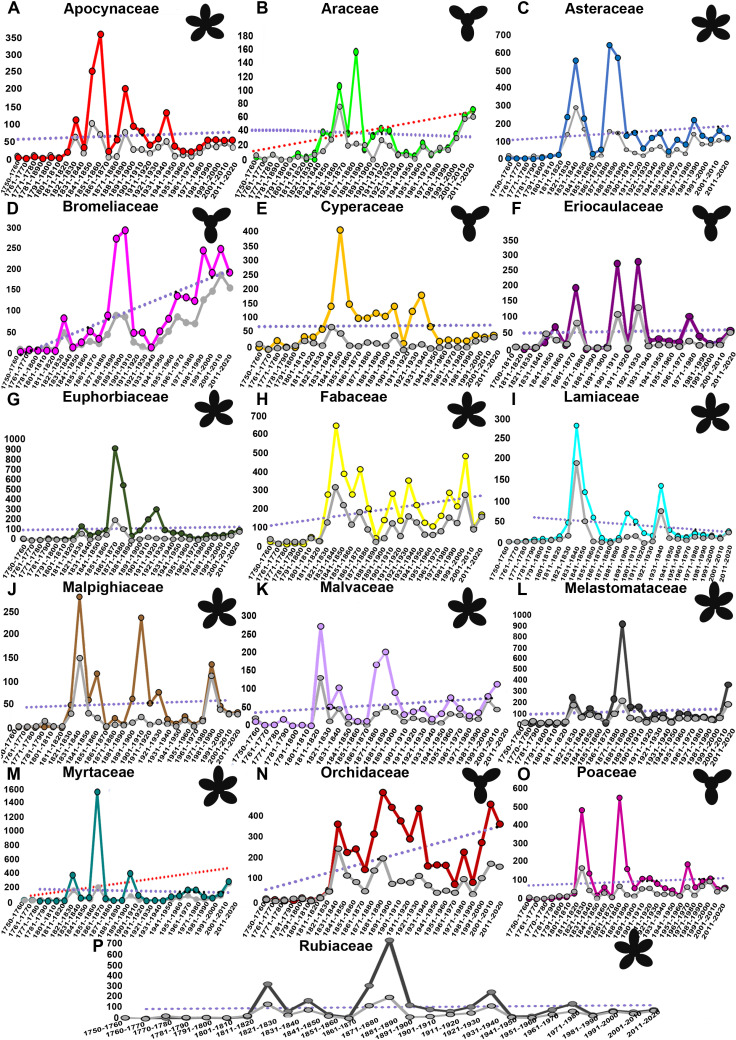
Species discoveries over time. **The number of angiosperm species published over time for the sixteen plant families with more than 500 species native to Brazilian territory. Graphics by family show the uneven distribution of newly described species of plants per decade. Colored lines represent the “all names” dataset, gray lines represent the “basionym” dataset. Violet lines represent the trend line for “all names,” while red dashed lines indicate the linear trend for basionym data, specifically for families where the trend differs between the datasets.** A. Apocynaceae; B. Araceae; C. Asteraceae; D. Bromeliaceae; E. Cyperaceae; F. Eriocaulaceae; G. Euphorbiaceae; H. Fabaceae; I. Lamiaceae; J. Malpighiaceae; K. Malvaceae; L. Melastomataceae; M. Myrtaceae; N. Orchidaceae; O. Poaceae; P. Rubiaceae.

**Fig 3:**
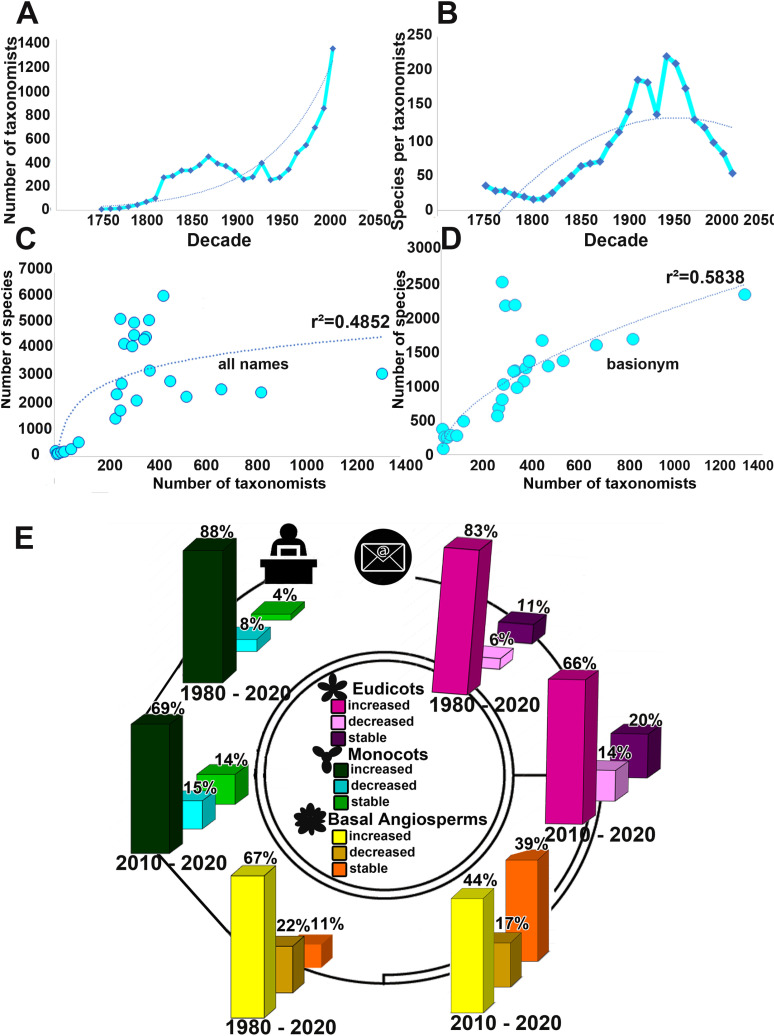
Taxonomic effort. **A**. Number of taxonomists of flowering plants active per decade. **B**. The number of species of flowering plants described from 1753–2020 divided by the number of taxonomists who described them. **C**. The regression line showing the relationship between the number of taxonomists per decade and the number of species described for angiosperms (r² = 0.4852) for ‘all names” dataset. **D**. The regression line showing the relationship between the number of taxonomists per decade and the number of species described for angiosperms (r² = 0.5838) for “basionym” dataset. **E**. Taxonomic effort per major clade, from survey data eudicots (shades of pink), monocots (shades of green) and basal angiosperms (shades of yellow).

**Fig 4:**
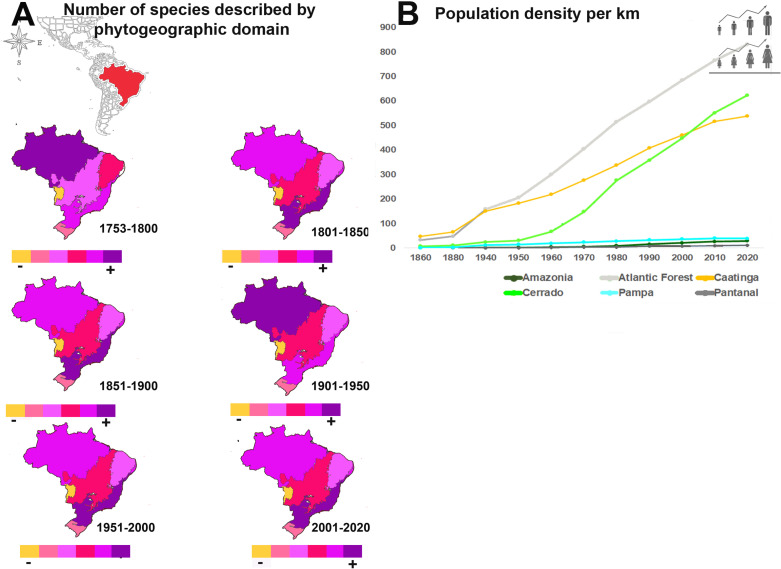
Data analysis by phytogeographic domains in the Brazilian territory: **A**. Number of species per major time interval and phytogeographic domain. **B**. Human population density per Km^2^ by domain over time.

**Fig 5:**
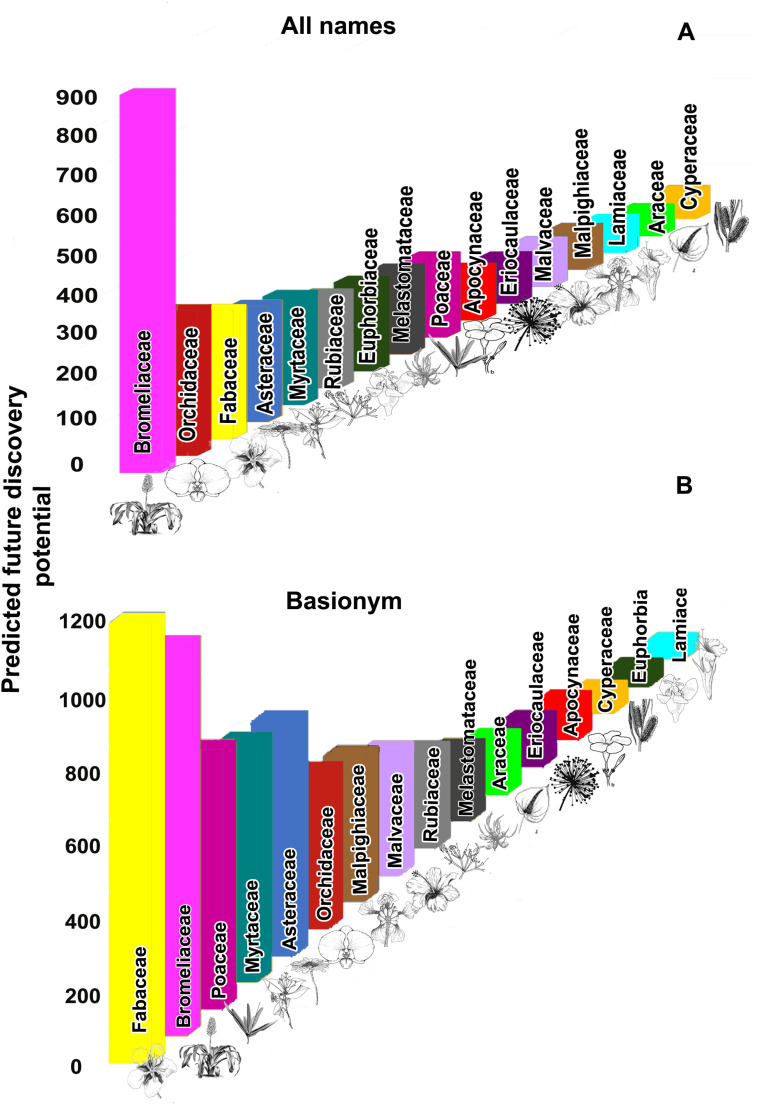
Predicted future discovery potential for angiosperm families containing more than 500 species. Top 10 angiosperm families with the highest potential for species discoveries. The bar height indicates the percentage of all future Brazilian angiosperm discoveries predicted to occur in the taxon. **A**. Families with the highest number of total discoveries based on all angiosperm names. **B**. Families with the highest number of total discoveries based on angiosperm basionyms.

**Fig 6:**
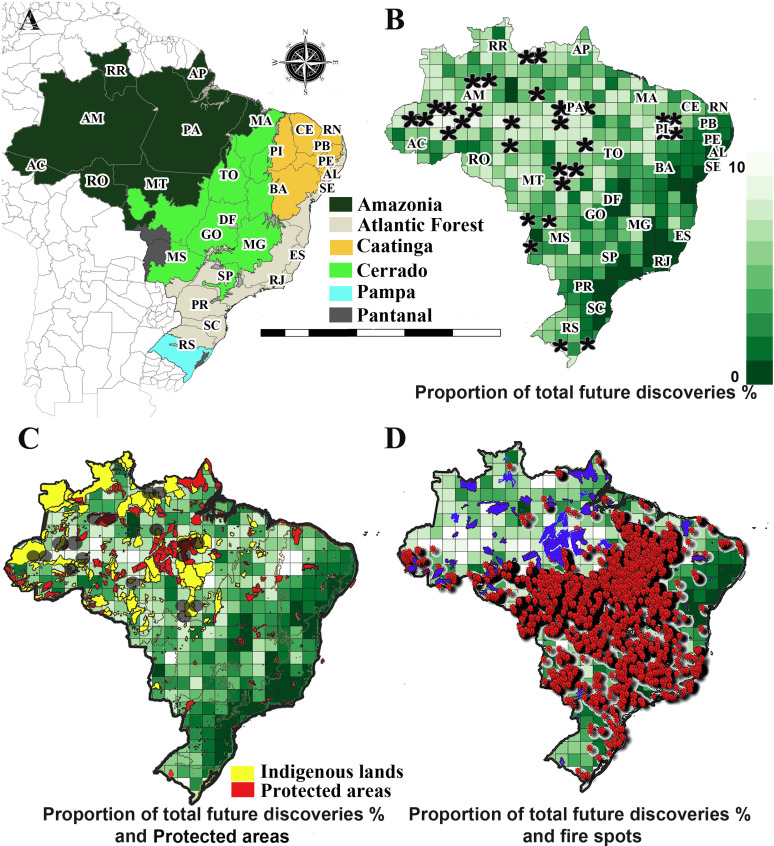
Variation in predicted discovery potential, and completeness of taxonomic inventories for angiosperms in Brazilian territory. **A**. Map of Brazilian territory with domains and states. **B**. Areas with highest potential of future discoveries for angiosperm across Brazilian phytogeographic domains. Variation across Brazilian phytogeographic domains, with colors showing discovery potential, standardized to ratio of discoveries from 0 to 10. White areas indicate regions that emerge as holding the greatest discovery opportunities, and dark green indicate the lowest opportunity. The flowers in black indicate the highest discovery potential. **C**. Areas with highest potential of future discoveries for angiosperm across Brazilian phytogeographic domains and protected areas. Black circles indicate areas with the highest discovery potential, superimposed on the distribution of Brazilian protected areas, defined here as the sum of Conservation Units (red) and Indigenous Lands (Terras Indígenas – TI, yellow). Note: In the Brazilian context, “protected areas” include both categories [43]. **D**. Areas with highest potential of future discoveries and fire spots. Protected areas, conservation units [45] in blue, and fire in red (PRODES – INPE 2023].

Figs 1–4b and 5 were generated using Microsoft Excel and subsequently edited in Adobe Photoshop to enhance visual clarity. Figs 4a, 6, and S1 were created through the overlay of spatial data (shapefiles) in QGIS and edited in Adobe Photoshop.

## Results

### Discovery pattern, discovery rate and number of taxonomists

For both ‘all names’ and ‘basionyms’, the pattern of angiosperm description between 1753 and 2020 started with an initial slow stage (Fig 1a), followed by a rapid ascent in the early 19th century to a ‘high plateau’ stage, followed by two sharp declines, one at the turn of the 20^th^ century with a partial recovery 1920-40 and the second between 1940 and 1950. Despite annual fluctuations, the current stage, from 1950 to the present day, is generally described as a “steady ascent’ with some fluctuation in “all names” and a clear uptick in the 21st century.

The analysis of habit data from BFG (Fig 1b), showed that the Amazonian domain has the highest percentage of tree species described, while the Atlantic Forest and the Pampa have the highest proportion of herb species described, and the Caatinga and the Cerrado have shrubs as their dominant species. When categorized by habit (Fig 1c, d), our two names’ datasets reveal similar trajectories for herbs and shrubs. Both all names and basionyms exhibit a peak around 1830, followed by a decline between 1920–1930, and a subsequent resurgence in the year 2000. Trees and lianas experienced a later peak around 1850, a decline in 1920, and have been on an upward trajectory in the number of described species since 2000. Herb and shrub species not known to science are probably most likely among future additions to the Brazil flora as they are further from the asymptote, showing more ascending trends. In contrast, trees and lianas exhibit a lower probability of future discovery, being closer to the asymptote and displaying less pronounced upward trends.

As with all published names for angiosperms analyzed together, when families are plotted individually (Fig 2), the same general pattern is seen. This pattern typically includes three stages: an initial slow stage of species description, rapid ascent to one or more peaks between 1801–1900, followed by a decline to or below the mean, and relatively steady or slightly increasing rates in recent decades. The families Apocynaceae, Araceae, Asteraceae, Eriocaulaceae, Malpighiaceae, Malvaceae, and Poaceae had more than one acceleration peak in name publication. In some families (e.g., Lamiaceae), such peaks are clearly mainly attributable to an underlying basionym peak, indicating periods when significant and lasting additions were made to Brazil’s taxonomic inventory for that group. However, for others (e.g., Myrtaceae, Poaceae) the highest peaks are not or barely underpinned by basionym peaks, suggesting periods of major remodelling resulting in many new names which did not stand the test of time. As for all angiosperms, the trend line representing only basionyms is very close to the trend line of “all names” from 1940 to 2020 for almost all families. But, in contrast to the all-angiosperm trends, for many families the two trend lines overlap, at least in part, over recent decades. Notable exceptions are Bromeliaceae and Orchidaceae. Several factors contribute to the different patterns seen in these families, the most obvious being the high number of infraspecific taxa described.

The sixteen angiosperm families with over 500 species native to Brazil had their highest peaks in the number of species described between 1800 and 1900 (Fig 2), a time when there were fewer taxonomists than in the current decade (see Fig 3a). On the other hand, most of Brazil’s plant diversity had not yet been described, facilitating the process of identification and description of new species. Using linear trend analysis of the ‘all names’ data, we observed that most families displayed a fairly constant number of names being published over time. However, three families, i.e., Bromeliaceae, Fabaceae, and Orchidaceae, stood out by exhibiting an ascending trend in publication of new names. This suggests a gradual increase in the number of new names published over the years, indicating a considerable distance from the asymptote. Using the basionym dataset, the linear trend lines differ only for the families Araceae and Myrtaceae, which show an upward trend in the publication of new species. The graphs reveal that in most families the numbers of new names published in recent decades have tended to be below the 270-year average for that family, notable exceptions include Araceae and Bromeliaceae. However, with the exception of Malpighiaceae, most families don’t show a declining trend over the past four decades: families exhibiting stable numbers (e.g., Apocynaceae, Rubiaceae) over that period are less frequent than those showing slight (e.g., Cyperaceae, Euphorbiaceae, Lamiaceae) or marked upward trends (e.g., Eriocaulaceae, Malvaceae, Myrtaceae). Other families show fluctuating numbers over the period.

Analysis of the trajectory of the number of taxonomists active in taxonomy of Brazilian angiosperms over the period from 1753 to 2020 (Fig 3a–b) reveals a clear exponential trend pattern. The observed exponential curve evidences a fairly consistent and accelerating increase in the number of scientists engaged in taxonomy over time. The mean number of angiosperm species described per taxonomist active per decade (Fig 3b) exhibits a polynomial pattern.

We found a positive relationship between the number of taxonomists per decade and both names datasets. This shows that a significant proportion of the variation in the number of reported species can be accounted for by changes in the number of taxonomists. The logarithmic regression line, characterized by a coefficient of determination r^2^ = 0.4852 for the dataset “all names” (Fig 3c), and a coefficient of determination r^2^ = 0.5838 for the dataset “basionym” (Fig 3d) indicates the moderate strength of this relationship. The p-value for “all names” is 0.0275, indicating that the relationship between the variables is statistically significant. In contrast, the p-value for “basionym” is 0.0000648, indicating a highly statistically significant relationship between the variables. The lower p-value in the “basionym” dataset suggests a stronger and more direct relationship between the number of taxonomists and species described, likely due to less variability and more precise data compared to “all names”.

### Taxonomists perceptions of taxonomic effort

According to the opinions of 83% and 88% of taxonomic experts on eudicots and monocots (Fig 3e), respectively, between 1980 and 2020 the taxonomic effort focused on their group increased. In the opinion of 6% and 8%, respectively, taxonomic effort decreased, and for 11% and 4% of eudicot and monocot experts respectively, taxonomic effort appeared stable over these four decades. In contrast, opinions among basal angiosperm specialists were more varied: 67% believed the taxonomic effort increased between 1980 and 2020, 22% felt it decreased, and 11% thought it remained stable.

When only the most recent decade, from 2011 to 2020, is considered, the proportion of taxonomists reporting an increase in taxonomic effort falls to 66%, 69%, and 44% of experts in eudicots, monocots, and basal angiosperms, respectively. A larger minority (14%, 15%, and 17%) of experts believe taxonomic effort decreased in their group, while 20%, 14%, and 39% of experts in eudicots, monocots, and basal angiosperms, respectively, believe that it stayed consistent over the last decade.

Evaluating the 16 families (Table 1; Fig 2) with more than 500 described species native to Brazil, we found interesting discrepancies between expert estimates of taxonomic effort and the actual number of species described per family. Most taxonomists stated that the taxonomic effort increased both over the last four decades and between 2010 and 2020, but taking into account the basionym dataset trend, the actual number of newly described species decreased for three families (Malpighiaceae, Malvaceae, and Rubiaceae) and stabilized for three families (Apocynaceae, Asteraceae, and Cyperaceae) in the last four decades, compared to the previous decades.

**Table 1:** Difference between estimated taxonomic effort and actual number of species described per period and per family. Abbreviations: Increased = I; Stable = S; Decreased = D.

Family	Estimate of taxonomic effort over in the last four decades	Real number of species described between 1980–2020	Estimate of taxonomic effort in the last decade	Real number of species described between 2010–2020
**Apocynaceae**	I	S	I	S
**Araceae**	I	I	I	I
**Asteraceae**	I	S	I	D
**Bromeliaceae**	I	I	I	D
**Cyperaceae**	I	S	S	I
**Eriocaulaceae**	I	I	S	I
**Euphorbiaceae**	I	I	I	I
**Fabaceae**	I	I	I	I
**Lamiaceae**	I	S	I	I
**Malpighiaceae**	I	D	I	D
**Malvaceae**	I	D	I	I
**Melastomataceae**	I	I	I	I
**Myrtaceae**	I	D	I	I
**Orchidaceae**	I	I	I	D
**Poaceae**	I	I	I	D
**Rubiaceae**	I	D	I	

Specialists in Eriocaulaceae assessed the taxonomic effort as stable over the last decade, despite the fact that the number of reported species for this family grew. Some specialists perceived a rise in taxonomic effort, even though the number of species described declined in their families, i.e., Bromeliaceae and Orchidaceae (see basionym trend Fig 2).

### Description by phytogeographical domain, opportunities for species discovery, and conservation of areas

Details of the number of species predicted, areas with the highest discovery potential, human population density by phytogeographic domain, and statistical fits, along with maximum–likelihood confidence intervals (CIs) of the estimates, are provided in Tables 2–4, Supporting Information, S4–S6 Appendices, S1 Fig and Figs 4–6.

**Table 2:** Summarizes estimates, Predictions of different species discovery models. Comparison of models, number of known and predicted species, and percentage of expected increase, of flowering plants, by the Brazilian phytogeographic domains. Number of currently known of native and endemic species per phytogeographic domain and predictions species (Asymptote, undescribed species) in different phytogeographic domains, along with parameter estimates and maximum–likelihood CIs, AIC (Akaike’s Information Criterion), AICc Weight, BIC (Bayesian Information Criterion) and Completeness Index. For cases where the Weibull Growth model did not converge to real values (BIC number, AIC weight, or asymptote value), these are indicated by a ‘__’ in the table. Models with the best fit for the evaluated database are highlighted in bold. The table includes different results for ‘Taxonomic effort’ based on the best-supported parameter estimations for the data.

Region	Model name	Species catalogued to date	Predicted/ species remaining to be described	Lower 95%	Upper 95%	AIC (Akaike’s Information Criterion)	AIC Weight	BIC (Bayesian Information Criterion)	Completeness Index
**Amazon**	**Gompertz 3P, all names**	11,903	**1403**	**748**	**2133**	**392,339**	**1**	**397,5223**	
**Amazon**	Logistic 3P, all names	11,903	488	–1325	469	432,9731	0	438,1564	
**Amazon**	Weibull Growth, all names	11,903	**_____**	**_____**	**_____**	**_____**	**_____**	**_____**	
**Amazon**	**Gompertz 3P, basionym**	11,903	**3512**	**2571**	**4729**	**344**	**1**	**348,9795**	
**Amazon**	Logistic 3P, basionym	11,903	817	224	1591	371,3059	0	376,4893	
**Amazon**	Weibull Growth, basionym	11,903	_____	_____	_____	_____	_____	_____	
**Amazon**	Taxonomic effort, all names	11,903	3205			563,8565	0,9955035	567,744	
**Amazon**	**Taxonomic effort, all names**	11,903	**1706**			**552,5408**	**0,993705**	**556,4283**	
**Amazon**	Taxonomic effort, basionym	11,903	2762			270	0,5	274,3081	
**Amazon**	**Taxonomic effort, basionym**	11,903	**4223**			**460**	**0,9999477**	**463,9657**	
**Amazon**	**Average**		**2711**	**2312**	**3198**				**81,44%– 78,81%**
**Amazon**	**Model Averaging**		**≈3086**		**≈3200**				**79, 42% – 78,81%**
**Atlantic Forest**	Gompertz 3P, all names	14,905	1107	–1473	3687	448,74259	5,60E–05	452,10776	
**Atlantic Forest**	Logistic 3P, all names	14,905	1341	1050	1632	436,75184	0,0225008	440,11701	
**Atlantic Forest**	**Weibull Growth, all names**	14,905	**1455**	**1189**	**1720**	**429,20966**	**0,9771534**	**432,57482**	
**Atlantic Forest**	**Gompertz 3P, basionym**	14,905	**234**	**–1151**	**2726**	**416**	**1**	**420,8427**	
**Atlantic Forest**	Logistic 3P, basionym	14,905	1008	–1385	2054	428,7049	0	433,8882	
**Atlantic Forest**	Weibull Growth, basionym	14,905	**_____**	**_____**	**_____**	**_____**	**_____**	**_____**	
**Atlantic Forest**	**Taxonomic effort, all names**	14,905	**578**			**336,5724**	**1**	**340,4599**	
**Atlantic Forest**	Taxonomic effort, all names	14,905	1172			566,6801	0,9995983	570,5676	
**Atlantic Forest**	Taxonomic effort, basionym	14,905	1925			479	0.6852662	482,7039	
**Atlantic Forest**	**Taxonomic effort, basionym**	14,905	**3148**			**466,2374**	**0,9941927**	**470,1249**	
**Atlantic Forest**	**Average**		**1354**	**941**	**2043**				**91,46% – 87,94%**
**Atlantic Forest**	**Model Averaging**		**≈1350,85**		**≈2043**				**91,69% – 87,94%**
**Caatinga**	Gompertz 3P, all names	4,781	536	–73251	74322	404,66951	0,0019087	408,03467	
**Caatinga**	Logistic 3P, all names	4,781	445	268	622	404,70649	0,0018738	408,07165	
**Caatinga**	**Weibull Growth, all names**	4,781	**577**	**443**	**710**	**392,16992**	**0,9885442**	**395,53508**	
**Caatinga**	**Gompertz 3P, basionym**	4,781	**831**	**61**	**5166**	**346,1972**	**1**	**351,38053**	
**Caatinga**	Logistic 3P, basionym	4,781	214	–357	2578	354,1448	0,0183362	359,32814	
**Caatinga**	Weibull Growth, basionym	4,781	**_____**	**_____**	**_____**	**_____**	**_____**	**_____**	
**Caatinga**	**Taxonomic effort, all names**	4,781	**3640**			**314,0847**	**1**	**317,9722**	
**Caatinga**	Taxonomic effort, all names	4,781	3191			525,2774	0,9999106	529,1649	
**Caatinga**	**Taxonomic effort, basionym**	4,781	**2825**			**396,515**	**0.9805329**	**400,4026**	
**Caatinga**	Taxonomic effort, basionym	4,781	2122			205,3618	0,9454991	209	
**Caatinga**	**Average**		**1968**	**1742**	**3085**				**70,84% – 60,78%**
**Caatinga**	**Model Averaging**		**≈1968,06**		**≈3093**				**70,84% – 60,71%**
**Cerrado**	Gompertz 3P, all names	12,025	579	463	696	384,73676	0,0450535	388,10192	
**Cerrado**	Logistic 3P, all names	12,025	558	434	682	389,35234	0,004482	392,7175	
**Cerrado**	**Weibull Growth, all names**	12,025	**630**	**525**	**734**	**378,63863**	**0,950428**	**382,0038**	
**Cerrado**	**Gompertz 3P, basionym**	12,025	**3**	**–1027**	**1021**	**403,01804**	**0,9982167**	**406,38321**	
**Cerrado**	Logistic 3P, basionym	12,025	**_____**	**_____**	**_____**	**_____**	**_____**	**_____**	
**Cerrado**	Weibull Growth, basionym	12,025	**_____**	**_____**	**_____**	**_____**	**_____**	**_____**	
**Cerrado**	Taxonomic effort, all names	12,025	524			541,348	1	545,2356	
**Cerrado**	**Taxonomic effort, all names**	12,025	**675**			**540,2072**	**1**	**544,0947**	
**Cerrado**	Taxonomic effort, basionym	12,025	605			370,0488	0,9990161	373,9363	
**Cerrado**	**Taxonomic effort, basionym**	12,025	**740**			**342,9918**	**0,9911365**	**346,8793**	
**Cerrado**	**Average**		**512**	**228**	**793**				**95,91% – 93,91%**
**Cerrado**	**Model Averaging**		**≈486**		**≈712**				**96,11% – 94,40%**
**Pampa**	Gompertz 3P, all names	2,578	167	103	230	354,44433	0,0691294	357,8095	
**Pampa**	Logistic 3P, all names	2,578	167	81	252	354,44433	0,0691294	357,8095	
**Pampa**	**Weibull Growth, all names**	2,578	**209**	**135**	**283**	**350,18812**	**0,5806143**	**353,55329**	
**Pampa**	**Gompertz 3P, basionym**	2,578	**83**	**–159**	**158**	**310,32261**	**0,9995591**	**313,68778**	
**Pampa**	Logistic 3P, basionym	2,578	151	–216	229	325,7781	0,0004402	329,14327	
**Pampa**	Weibull Growth, basionym	2,578	195	–270	315	338,82804	6,46E–07	342,19321	
**Pampa**	**Taxonomic effort, all names**	2,578	**596**			**235,847**	**1**	**239,7345**	
**Pampa**	Taxonomic effort, all names	2,578	588			277,1137	1	281,0012	
**Pampa**	Taxonomic effort, basionym	2,578	391			302,1668	1	306,0544	
**Pampa**	**Taxonomic effort, basionym**	2,578	**115**			**282,1486**	**1**	**286,0361**	
**Pampa**	**Average**		**251**	**171**	**288**				**91,12% – 89,95%**
**Pampa**	**Model Averaging**		**≈285,22**		**≈361**				**89,94% – 87,71%**
**Pantanal**	Gompertz 3P, all names	1470	135	94	176	326,97209	0,4762383	330,33725	
**Pantanal**	Logistic 3P, all names	1470	106	–487	700	334,98534	0,008665	338,35051	
**Pantanal**	**Weibull Growth, all names**	1470	**135**	**95**	**175**	**326,95644**	**0,4799784**	**330,32161**	
**Pantanal**	**Gompertz 3P, basionym**	1470	**11**	**–26**	**59**	**219,3711**	**1**	**224,5544**	
**Pantanal**	Logistic 3P, basionym	1470	43	36	61	233,4065	0,0008951	238,58982	
**Pantanal**	Weibull Growth, basionym	1470	65	–87	105	250,1418	2,08E–07	255,3251	
**Pantanal**	Taxonomic effort, all names	1470	251			327,1492	0.9999997	331,0367	
**Pantanal**	**Taxonomic effort, all names**	1470	**116**			**320,8431**	**0,9999964**	**324,7307**	
**Pantanal**	Taxonomic effort, basionym	1470	255			146,9947	0,9999999	150,8822	
**Pantanal**	**Taxonomic effort, basionym**	1470	**157**			**135,8462**	**0,9999978**	**139,7337**	
**Pantanal**	**Average**		**105**	**86**	**127**				**93,33% – 92,04%**
**Pantanal**	**Model Averaging**		**≈167**		**≈186**				**89,79% – 88,76%**
**Angiosperms**	Taxonomic effort, all names		11749			665,6512	0.927482	669,5387	
**Angiosperms**	**Taxonomic effort, all names**		**12862**			**664,1647**	**0,9999999**	**668,0522**	
**Angiosperms**	**Taxonomic effort, basionym**		**10107**			**652,7798**	**0,9999996**	**656,6673**	
**Angiosperms**	Taxonomic effort, basionym		6823			645,3386	0,9664978	649,2261	
**Angiosperms**	**Average/** **Model Averaging**		11485						

**Table 3:** Predicted new species per Brazilian phytogeographic domains using different approaches. Basionym: predicted number of species, considering only the basionyms. Basionym, upper 95%: upper 95% confidence interval for the predicted number of species considering basionyms. All names: predicted number of species considering all names (including synonyms). All names, upper 95%: upper 95% confidence interval for the predicted number of species considering all names. Taxonomic effort, basionym: number of species accounting for taxonomic effort when basionyms are taken into consideration. Taxonomic effort, all names: number of species accounting for taxonomic effort considering all names. Sum of the averages: sum of the average predictions across all models. Upper 95%: upper 95% confidence interval for the sum of the average predictions. Model averaging is the weighted average of predictions from different models. Model Averaging, Upper 95%: Upper 95% confidence interval for the model–averaged predictions. Model Averaging Media: Model Averaging + Model Averaging, Upper 95% confidence interval for the model–averaged predictions per phytogeographic domain. The rows represent different biomes, with the final row providing the total counts across all biomes.

Phytogeographic domain	TOTAL PER DOMAIN										
	Names				Taxonomic effort				Model		
	Basionym	Basionym, Upper 95%	All names	All names, Upper 95%	Basionym	All names	Sum of the averages	Upper 95%	Averaging	Averaging, Upper 95%	Averaging Media
Amazon	3512	4729	1403	2133	4223	1706	2711	3198	3086	3200	3143
Atlantic Forest	234	2726	1455	1720	3148	578	1354	2043	1350,85	2043	1697
Caatinga	831	5166	577	710	2825	3640	1968	3085	1968,06	3093	2531
Cerrado	3	1021	630	734	740	675	512	793	486	712	599
Pampa	83	158	209	283	115	596	251	288	285,22	361	323
Pantanal	11	59	135	175	157	116	105	127	167	186	177
**Total average**	**4674**	**13859**	**4409**	**5755**	**11208**	**7311**	**6901**	**9534**	**7343,13**	**9595**	**8470**

**Table 4:** Summary of regression analysis for the relationship between the number of species and the human population density, as well as the number of taxonomists and the number of species across different phytogeographic domains. Within–range human population density at the year of species’ description. The type of regression that best fits each relationship is indicated, along with the equation and the R^2^ value.

Phytogeographic domains/ Time	Type of Regression (Human population density *vs*. Number of Species)	R2 (Density *vs.* Species)	Type of Regression (Number of Taxonomists *vs*. Number of Species)	R^2^ (Taxonomists *vs.* Species)
**Amazon, all names (1753–2020)**	Logarithmic	0.9109	Logarithmic	0.589
**Atlantic Forest, all names (1753–2020)**	Logarithmic	0.9327	Logarithmic	0.6224
**Caatinga, all names (1753–2020)**	Logarithmic	0.9257	Exponential	0.6456
**Cerrado, all names (1753–2020)**	Logarithmic	0.8347	Exponential	0.7402
**Pampa, all names (1753–2020)**	Logarithmic	0.9541	Logarithmic	0.6534
**Pantanal, all names (1753–2020)**	Logarithmic	0.8401	Logarithmic	0.6393
**Amazon, basionym (1753–2020)**	Logarithmic	0.9701	Linear	0.6227
**Atlantic Forest, basionym (1753–2020)**	Potential	0.9433	Linear	0.6678
**Caatinga, basionym (1753–2020)**	Linear	0.9529	Linear	0.7383
**Cerrado, basionym (1753–2020)**	Logarithmic	0.8839	Linear	0.6632
**Pampa, basionym (1753–2020)**	Logarithmic	0.9777	Logarithmic	0.7557
**Pantanal, basionym (1753–2020)**	Logarithmic	0.8939	Logarithmic	0.5927
**Amazon, basionym (1960–2020)**	Polynomial	0.5917	Linear	0.1073
**Atlantic Forest, basionym (1960–2020)**	Exponential	0.9003	Linear	1
**Caatinga, basionym (1960–2020)**	Logarithmic	0.862	Logarithmic	0.8223
**Cerrado, basionym (1960–2020)**	Potential	0.907	Logarithmic	0.9413
**Pampa, basionym (1960–2020)**	Potential	0.3137	Linear	0.4014
**Pantanal, basionym (1960–2020)**	Logarithmic	0.6631	Logarithmic	0.6037

The Amazon and Atlantic Forest domains consistently occupy the leading positions in the number of newly described species, alternating between first (Fig 4a in purple) and second place (Fig 4a in violet) since 1753. Patterns are the same for the two name datasets at this scale. Examining the number of species described by domain and decade, Amazonia led in species descriptions during the periods 1753–1800 and 1901–1950, with the Atlantic Forest leading in all the remaining decades.

Over the past three centuries, the Cerrado has consistently held the third position in the number of described species per domain, except for the period 1753–1800 when Caatinga ranked third, falling to fourth position thereafter. Pampa and Pantanal have consistently held the fifth and sixth positions, respectively, since 1753.

The regression analysis shows a positive correlation between human population density and the number of described species in all domains (Table 4). The best fit for the basionym dataset ranges from r^2^ = 0.8839 in Cerrado to r^2^ = 0.9777 in Pampa. The corresponding analysis for all names and human population density also showed positive correlations in all domains, however the *R*^*2*^ values were lower in each case.

The effectiveness of taxonomic effort as a predictor of species discovery and description varies not only over time but also between phytogeographical domains. Although the regression analysis shows that taxonomic effort, as measured by the number of taxonomists involved in publishing new names per decade, and the number of newly described species are positively correlated in all domains, ranging from r^2^ = 0.5927 in the Pantanal, to r^2^ = 0. 7557 in the Pampa to basionym dataset (Table 4), their r^2^ values are generally lower than those of population density. These findings over the long term suggest that human population density may be a more reliable indicator for predicting future description of as yet undescribed species than taxonomic effort. The higher coefficient of determination *R*^*2*^ values for population density across all domains indicates its potential as a better predictor in this context.

However, from 1960 to 2020 (Table 4), the results show a notable shift in the relative importance of factors predicting species discovery. Human population density has lost some of its predictive strength, particularly in Amazonia and in the Pampa domain compared to 1753–2020. In contrast, with the exception of Amazonia and Pampa, taxonomic effort has increased in explanatory power across domains for the period 1960–2020, compared to 1753–2020. The combined effect of these changes is that for the period 1960–2020, R² values for taxonomic effort surpassed those for population density in the Atlantic Forest, Cerrado, and Pampa domains, based on basionyms.

We compiled the full taxonomic data of currently accepted species of angiosperms in Brazil, to model the probability of species description, and predict the number of species remaining to be described in the future. When evaluating the uncertainty between different models and weighting their predictions based on how well they fit the data, we found that according to Akaike’s Information Criterion (AIC), AICc Weight, and Bayesian Information Criterion (Table 2; Supporting Information S6 Appendix), the most reliable estimates were provided by Gompertz 3P, mainly for the dataset basionym and Weibull growth for the dataset all names. The least reliable estimates were obtained with the Logistic 3P model. In addition, for some datasets the Weibull growth model did not converge to meaningful values.

Using a model averaging approach based on Akaike’s Information Criterion to predict the asymptotic number of species of each domain and for all angiosperms, the statistical model predicted 3086–3200 species remaining to be described in the Amazon, 1351–2043 for the Atlantic Forest, 1968–3093 for the Caatinga, 486–712 for the Cerrado, 285–361 for the Pampa, 167–186 for the Pantanal, and 11485 for all angiosperm species (Tables 2 and 3) to approach the asymptote.

Using the comparison of the actual inventoried number of species with that predicted by the models to calculate the completeness of the taxonomic inventory in each phytogeographic domain, we found that the catalog of Brazilian angiosperms is 19–23% incomplete, ranging from 4% incomplete in the Cerrado to 39% incomplete in the Caatinga. The results show that while the catalogs of Cerrado, Pampa, and Pantanal are closer to their asymptotes, the numbers of angiosperm species described in Amazonia, Atlantic Forest, and Caatinga may increase by 21.2%, 12.1% and 39.9% respectively, to approach the asymptote.

There are interesting discrepancies between the results of the different models (Tables 2 and 3;Supporting Information S6 Appendix), indicating that they do not always capture the same patterns or estimate the same number of unknown species. A congruent picture emerges from evaluating the model averaging, the sum of the averages by phytogeographical domain, and the total number of angiosperms.

Moreover, the results of 10107 (basionym-based) and 11485 (model averaging) undescribed species from the analysis using the “angiosperms” dataset (Table 2) corroborate the total sum of the phytogeographic domains and averages of the models, which estimated 8096–9595 undescribed species (Table 3). This further reinforces the reliability of the multiple model predictions and highlights the substantial number of species yet to be described, emphasizing the need for continued taxonomic research in these biodiverse regions.

Analysis of temporal description patterns for major families of angiosperm species, including their accumulation curves and the fit of their nonlinear models, and in particular the difference between the results from the basionyms dataset (Fig 5a; more details at: Supporting Information, S4 and S5 Appendices) and those from the “all names” dataset (Fig 5b), reveals that some families stand out as having significantly higher discovery potentials. Specifically, Fabaceae, Myrtaceae, and Poaceae have three times more species predicted in the model using only the number of basionyms as in the model using all names. Conversely, the Euphorbiaceae presents the number of predicted species three times smaller when using the basionym model compared to the model that includes all names.

Estimates of the numbers of undescribed species for individual families containing more than 500 species native to Brazil revealed that in both analyses (basionym only and all names), the five families Asteraceae, Bromeliaceae, Fabaceae, Myrtaceae, and Orchidaceae consistently rank among the top six families with the highest number of predicted species (Fig 5) or greatest potential for discovery. In contrast, Araceae, Cyperaceae, Eriocaulaceae, and Lamiaceae are consistently among the six major families with the lowest number of predicted species to be described (Fig 5). Notably, some of the families with greatest discovery potential, such as Asteraceae, Bromeliaceae, and Orchidaceae have experienced a decline in the number of species descriptions in the last decade (Fig 2).

According to our models, of the 428 cells, we identified 30 cells with the highest discovery potential (Fig 6b–d; Supporting Information S1h Fig). Of the 21 cells in Amazonia with high discovery potential, eleven are in the state of Amazonas, six are in the state of Pará, and four are in Mato Grosso. In Caatinga, the four cells with the high discovery potential are contiguous, forming a square in the state of Piauí. The Cerrado encompasses just half of one high discovery potential cell, located in the state of Mato Grosso, this is the only cell of the 30 that is divided between two phytogeographical domains, the other half being in Amazonia. The Pantanal has three high discovery potential cells, two in Mato Grosso and one in Mato Grosso do Sul. The two high discovery potential cells in the Pampa are in the southwest and southeast of Rio Grande do Sul. None of the 30 cells with highest discovery potential are located in the Atlantic Forest.

Most of the areas with the highest discovery potential identified in the current study (Fig 6b–d), do not overlap with PAs [45]. Of the six phytogeographic domains, only in Amazonia are PAs (highlighted PAs in black circles in Fig 6c) found within cells with highest discovery potential. In Amazonia, six of the 21 cells identified as highest discovery potential overlap with one or more PAs: three overlap with full protection areas, Parque Nacional do Jaú (Parna do Jaú), Parque Nacional da Amazônia (Parna da Amazonia), and Estação Ecológica Terra do Meio (ESEC da Terra do Meio); and four overlap with sustainable use areas, Reserva Extrativista Unini (RESEX Unimi situated on the lower Rio Negro in Amazonas), Reserva Extrativista Médio Juruá (RESEX Médio Juruá), Reserva Extrativista Rio Iriri (Resex Rio Iriri) and Flona de Tefé (Floresta Nacional de Tefé).

The areas with the highest probability of discoveries in Caatinga, Cerrado, Pantanal and Pampa do not have any full protection PAs or sustainable use areas (Fig 6c). Thus, in total, only 20% of the areas with the highest potential for future discovery of undescribed angiosperm species overlap with PAs and all of these are in Amazonia.

However, when indigenous lands are considered, the scenario is brighter; 15 of the 30 areas with the highest discovery potential overlap with areas of indigenous lands (highlighted in black circles in Fig 6c). In Amazonia, 12 of the 21 identified areas with the highest discovery potential overlap with indigenous lands. These overlap areas are mainly located in the state of Amazonas in: Vale do Javari (the second-largest indigenous reserve in the country in the extreme west of Amazonas), Kanamari do Rio Juruá, Rio Biá, Hi-Merimã, Banawá, Camadeni, Catipari/Mamoria, Água Preta/Inari, Guajahã, Seruimi/Mariene, Tumiã, Alto Sepatini, Acimâ, Apurinâ do Igarapé Mucuim, and Andirá-Marau. In the state of Pará several areas of highest discovery potential also overlap with indigenous lands: Trombetas/Mapuera, Kaxuyana-Tunayana, Andirá-Marau, Cachoeira Seca, and Trinchera Bacaja, and in Mato Grosso such overlap occurs with Kawahiva do Rio Pardo and Parque do Xingu.

Thus, according to our models, approximately 80% of the areas predicted to be rich in undescribed angiosperm species do not overlap with protected areas (PAs) at all, but 50% are inside indigenous lands. The as yet unknown species are concentrated in rare, difficult-to-access locations within domains that experience high levels of habitat destruction and fire (Fig 6c–d).

## Discussion

Determining the total number of species is central to many biological analyses, driven by scientific curiosity about life’s diversity and the need to establish benchmarks for assessing biodiversity decline, a crucial underpinning for conservation efforts [8,9,10]. Additionally, understanding species diversity is vital for identifying areas of high conservation priority and implementing effective strategies to protect biodiversity [6].

Integrating historical biodiversity data with current information offers a more holistic assessment of overall diversity and assists in identifying potential priority areas for conservation. In this study, we integrate historical and current biodiversity data across 267 years (1753–2020), analyzed for the first time, to offer a comprehensive estimate of Brazil’s angiosperm diversity and understand the patterns of species discovery across the phytogeographical domains.

### Biological and Socioeconomic contex

Brazil is widely recognized as one of the most diverse countries in terms of vascular plant species [20,21,23]. Of its six phytogeographic domains, two are recognized as global biodiversity hotspots, the Cerrado and the Atlantic Forest [46,47] and these domains are also the most-studied and best-represented in terms of numbers of preserved specimens [21,25,26]. In contrast, Amazonia remains one of the least–explored forests within the Americas, being underrepresented in biodiversity databases and specimen repositories [25, 26, present work]. Further complicating this scenario, anthropogenic alterations have the potential to erase tranches of Amazonia’s biodiversity even before we gain an understanding of the yet-to-be-identified species inhabiting this expansive region. This lack of knowledge and documentation poses a significant challenge for conservation efforts in Brazilian Amazon. Without a comprehensive understanding of the biodiversity present, it is difficult to effectively prioritize and protect threatened species and ecosystems.

The pace of discovery and description of angiosperm species in Brazil has fluctuated, exhibiting peaks and declines over time. These fluctuations are intricately linked to historical, socioeconomic, and political factors. For instance, corroborating trends observed in species discovery rates for other taxa and regions [e.g., 48, 49], rates of description of Brazilian angiosperms faced significant declines during the two World Wars (see Fig 1a; Fig 2).

Approximately 90% of Brazilian research is conducted in public universities, with the main locus of this production being *stricto sensu* postgraduate programs [50]. Institutionalized academic research activity in Brazil commenced in 1951 with the consolidation of the Brazilian National Council for Scientific and Technological Development, *Conselho Nacional de Pesquisa* (CNPq), and the *Coordenação Nacional de Aperfeiçoamento de Pessoal de Nível Superior* (CAPES). Data from the *Coordenação de Aperfeiçoamento de Pessoal de Ensino Superior* (CAPES/MEC) reveals a doubling of the number of students from the 1990s to 2000. Charting the timeline, the expansion of research in Brazil is closely linked to the integration of this activity into *stricto sensu* postgraduate studies [51] and the increase in the number of graduate programs in ecology, zoology, and botany from 2004 to 2013 [52]. Similarly, numbers of taxonomists in Brazil, and consequently, the number of described species, increased significantly from 1970 onwards, with the number of taxonomists increasing rapidly since 1990 (Fig 1a; Fig 3a).

Likewise, investment in research in the field of biological sciences experienced a significant increase, ranking highest among areas that received the most resources in the years 2011, 2013, and 2014, with many projects approved specifically in biodiversity [51, 52, 53]. Among them, we can highlight two significant projects that have contributed to the description of plant species in Brazil: the Plants of Brazil: Historic Rescue and a Virtual Herbarium for Knowledge and Conservation of the Brazilian Flora – Reflora” (“Plantas do Brasil:Resgate Histórico e Herbário Virtual para o Conhecimento e Conservação da Flora Brasileira – Reflora” in 2010 [54] and (PROTAX, Programa de Apoio a Projetos de Pesquisas para a Capacitação e Formação de Recursos Humanos em Taxonomia Biológica), launched in 2005 by the Brazilian National Council for Scientific and Technological Development (CNPq) [55]. The increase in the number of species described in the decade ending 2020 (Fig 1a) is likely attributable to enhanced funding, coinciding with a rise in the number of students in postgraduate courses. Conversely, we observed a decline in funding for taxonomy, botanical, and mycological sciences from 2019 to 2022 [21,52,53]. The scenario between 2019 and 2022 was perverse, with cuts in research funding as well as inefficiency in resource allocation, with effects that may be manifest in the short term. The absence of funding has led to a reduction in field collection efforts and a decrease in the number of students in postgraduate programs (https://sucupira–beta.capes.gov.br/sucupira4/), potentially resulting in a decrease in the number of species described, which may become evident in the coming years. Without adequate resources, researchers are struggling to maintain collection efforts and support postgraduate programs [56].

Two factors have kept Brazil at the forefront of newly described plant species globally and maintaining a constant pace of description. First, the country presents an exceptional richness of angiosperm species. Secondly, it benefits from a substantial number of professional taxonomists (Fig 3). However, the trend lines for species description in recent decades for some families with greatest species diversity (Fig 2) are showing a concerning decrease. This indicates a divergence in the trajectory, where the number of taxonomists has increased while the number of described species per taxonomist, has declined over time in Brazil. The decline in species descriptions may be attributable to the difficulty in accessing remote and unexplored regions of Brazil, where some of the areas identified in our results as likely to harbor undescribed species are located. Alternative explanations for this drop in species descriptions may include reduced funding for taxonomic research, a shift in scientific focus, or the increased difficulty in identifying and describing new species as the most widespread, conspicuous and distinctive species may already have been documented. Advances in technology and scientific methods might have led to a more rigorous and time–consuming process of species identification

Furthermore, adequately understanding a newly discovered species is a lengthy process that requires significant knowledge accumulation, it often takes decades between the collection of the first specimen and the publication of the species name [see more discussion in 57]. Therefore, the interval between the first collection and the description of the species may also be reflected in the graphs. It is important to note that the process of species description is not only time-consuming but also requires expertise in taxonomy, morphology, and evolution. Without proper documentation and research, many species could go unnoticed or misidentified.

### Undescribed diversity estimates, comparisons and limitations

By analyzing data from 1752 to 2020, we found human population density to be a strong predictor of species description rates over the centuries. This finding is supported by the present study and corroborated by previous studies, such as the analysis of anurans from the Brazilian Cerrado [35] and terrestrial vertebrate species [14], which indicate that the average description date was positively correlated with human population density in geographical space in Brazil.

Regression analysis (Fig 3c, d and Table 4) indicates that variations in the number of active taxonomists can account for half or more of the variation in the numbers of species descriptions, with the explanatory power varying among the phytogeographical domains. This suggests that taxonomic effort has a moderate but significant impact on the observed trends in species descriptions. On the other hand, the density of the human population over time can account for 90% of the variance in the number of described species. Consistent with our results, a recent analysis provides evidence that logistics, i.e., accessibility and distance to research facilities, and human influence factors explained 64% of the variation in ecological research probability in Amazonia [27].

Data from the asymmetric history of human settlement across phytogeographical domains over time suggest that population density rather than taxonomic effort may have contributed more to the rise of angiosperm descriptions over the past 250 years. However, when analyzing the shorter, more recent time period, taxonomic effort was found to surpass population density as a predictor of species descriptions in Atlantic Forest, Cerrado, and Pampa. The Amazon is a notable exception, although the explanatory power of human population density is considerably reduced in recent decades, it continues to be a more relevant factor than taxonomic effort for species description in this domain. These changes in predictive value over time merit further investigation and may be associated with decreases in the mean range size of species being newly described from the different regions: human population density estimates based on relatively small range sizes may be more variable and less likely to be correlated with the socioeconomic level of the broader region in which the species occurs.

Among our results the Amazon and the Caatinga stand out as having the highest predicted number of undescribed species, suggesting these regions have the greatest predicted undescribed biodiversity. This indicates that, while individual models may vary in their predictions, considering broader patterns and averages provides a more consistent and reliable estimate. Despite the individual model discrepancies, the overall trends suggest that the Amazon and the Caatinga are priority regions for taxonomic exploration and conservation efforts. The congruence of our results, encompassing both model outcomes and analyses of taxonomic inventory completeness, identified gaps, and density, highlights the value of using multiple approaches to gain insights into the distribution of unknown species. This approach helps to guide future research efforts effectively by identifying key areas with high biodiversity that warrant further investigation (Fig 6, Supporting Information, S1g–h Fig, shows the future discovery potential, white represents the highest discovery probability).

According to our models, there are approximately 7343 – (- 9595, upper 95% confidence interval), native angiosperm species still awaiting description in Brazil, i.e., the catalog may be 19–23% incomplete. Some 4% to 39% of the angiosperm species remains unknown to science per domain, with Amazonia and Caatinga offering the greatest potential for new species description. Considering the pace of species discovery over the past 20 years (Fig 1a–c), it is projected to take around ~48 years to describe all these species. Thus, if trends in description rate remain continuous for the next 30 years, Brazilian angiosperm richness is expected to remain growing until 2072, when it approaches the asymptote of cataloging. However, it is important to note that external factors such as habitat destruction and climate change could impact the rate of species discovery and potentially alter this projection, as well resourcing factors such as support for field collections, collections management and digitization.

Importantly, we developed models based on the description dates of currently accepted Brazilian angiosperm species, operating under the assumption that the taxonomic record remains stable. Nevertheless, taxonomic revisions, which consider both morphological and molecular evidence, have the potential to split (add) or synonymize (remove) species, thereby altering the projected rates and forecasts of undiscovered species. We recognize this as an important limitation to our work; nonetheless, mitigating the influence of taxonomic factors will consistently pose challenges in any comparative study [10].

Taking a broader perspective, it is important to acknowledge that our predictions are a direct reflection of past description processes and their influences, and any future interpretations must consider these inherent limitations. Despite efforts for taxonomic standardization and stability, species also constitute scientific hypotheses that are subject to review, refuted, or revalidation. In addition, the species concept has, for some taxa, changed considerably through time, including the changing trends of splitting or lumping species. As Bebber et al., [58] observed “the strong influence of unpredictable variations in the discovery process makes these data unreliable in estimating total species numbers”. In other words, we can use different attributes and models in an attempt to estimate total species numbers, but unpredictable variations decrease the accuracy of the results, and this possibility must be considered when interpreting the data.

Seeking to corroborate our results, we found no publication to date that incorporates information from all the angiosperms recorded in the six Brazilian phytogeographic domains and estimates numbers of species remaining to be described from each. However, we found elements of certain published studies which are qualitatively or quantitatively equivalent to ours, enabling useful comparisons.

In a study focused on estimating numbers of threatened plant species awaiting description in Brazil, Pimm et al. [59] used a subset of the angiosperm species then recorded from Brazil to estimate the likely increase in endemic species numbers in each of the five administrative regions of Brazil and found NE Brazil to be the region with by far the greatest potential for description of endemic new species (49% increase projected), while Southern, Northern and West-Central Brazil ranked 2^nd^ to 4^th^ by small margins (predicted to increase by 25, 24 and 21% respectively) and Southeastern Brazil ranked last (endemic flora predicted to increase by just 9%). Our results, based on Brazil’s phytogeographic domains rather than political regions, are surprisingly consistent with their results in some respects. For example, our results for Caatinga, a vegetation type confined to Brazil and almost exclusively to NE Brazil, indicate an expected increase of 39% in total species recorded (vs 49% endemic species for NE Brazil in Pimm et al., [59]) while our results for Brazilian Amazonia, an area largely coincident with the Northern Brazil administrative region, suggest an expected increase of 21% in recorded species (vs 24% endemic species for N Brazil in Pimm et al., [59]). Further south in Brazil the congruence between boundaries of regional and phytogeographic domains is much reduced, such that cross-study comparisons are arguably less meaningful, though the Pimm et al., [59] estimate of 9% endemic new species yet to be described for SE Brazil is comparable to our estimate of 12% for the Atlantic Forest.

As part of a global study of the Linnean and Wallacean shortfalls for plants [36] estimated numbers of species yet to be described from each of Brazil’s six administrative regions, ranged from 58 species in South Brazil to 148 in SE Brazil, and a Brazil-wide estimate totalling 478 additional species, with a 95% CI of 424–560 species. These surprisingly low estimates of numbers of undescribed species suggest that knowledge of the Brazilian angiosperm flora is > 98% complete and are not only an order of magnitude smaller than our results, but also far smaller than the documented (actual) rates of description of new species for the Brazilian flora in the 21st century (our study) and, more specifically, over the period 2020–2023 inclusive (ipni.org).

### Conservation significance and implications

Our findings suggest that around 80% of areas with high discovery potential do not overlap with protected areas (Fig 6c). While some areas with the highest probability of discovery in the Amazonia are partially superimposed with the PAs, we identified that Caatinga does not have any high discovery potential cells which overlap with full protection PAs. Although approximately 19% of the Brazilian territory falls within protected areas, including both full protection (6.37%) and sustainable use (12.29%) areas [45], the distribution of PAs across the landscape is noticeably uneven, as illustrated in Fig 6. A significant proportion of PAs is concentrated in Brazilian Amazonia (~28%, comprising 9.79% full protection areas and 17.87% sustainable use areas). In contrast, the Caatinga, encompassing only 7.5% of protected territory, has merely 1.3% under full protection [45,60]. Moreover, historically, the Caatinga has received comparatively less attention from public administration and the scientific community due to a twentieth-century conservation focus on preserving ‘rainforests’ [60,61].

Caatinga, Brazil’s largest and most diverse Seasonally Dry Tropical Forest [62], is highly vulnerable to human disturbance. There is significant concern that many plant species may go extinct before being described or fully understood [63,64]. Our results indicate that without sufficient public and scientific attention, coupled with conservation actions, there is a significant risk of these undescribed species disappearing. Such a loss not only represents a missed opportunity but also entails the forfeiture of unique evolutionary histories before their proper description and recognition.

These results highlight the imperative for expanding the coverage of PAs to ensure the effective protection of angiosperm species, especially in the Caatinga. This region currently lacks essential actions and investments for its preservation. The Caatinga is notably sensitive to human interference and the impacts of global climate change. A recent analysis focused on the Caatinga concluded that Brazil is significantly falling short of meeting the Aichi Target 11 concerning coverage, ecological representation, and effective management [60].

However, would PAs prove suffice for the preservation of Brazil’s angiosperm species? Over the span of approximately three centuries, or 267 years of collecting and describing angiosperm species in the Amazon (1753–2020), approximately 55% of the territory has been explored. Surprisingly, ~ 45% (1,888,624 km2) of the Brazilian Amazon territory lacks collected specimen of native and endemic angiosperm and even for many known species, there are few geographic records [25, 26, present work]. In stark contrast, over just 35 years (1988–2022), deforestation has claimed approximately 500,000 km^2^, with an average annual deforestation rate of 13,766 km^2^ (PRODES – Amazônia, INPE 2023; see S7 Appendix). This annual deforestation rate is twice the area covered by botanical collections per year over the course of 267 years. These data underscore the alarming pace of deforestation in Brazilian Amazonia, affecting both well–known and undocumented areas. It is evident that this pace far exceeds the capacity for species discovery and subsequent description. Thus, the main challenge to completing the taxonomic catalog and preserving Brazilian biodiversity is, undeniably, time.

The rapid loss of natural vegetation underscores the value of PAs, i.e., conservation units, in Brazil, designated to safeguard regions with established biological significance. But, unfortunately, as seen on the map (Fig 6d), not even Brazil’s PAs are immune to the threats of fire and deforestation, highlighting an unprecedented extinction risk. When deforestation occurs in undocumented areas, we lose the chance not only to know which unique species are present but also to pinpoint their attributes.

Moreover, although PAs in Brazil could play a key role in safeguarding many angiosperm species from extinction, coverage of angiosperm distributions by PAs remains incomplete. Recent studies show coverage of endemic angiosperm species distributions by PAs is not sufficient for effective protection with the current portfolio of Brazilian federal protected areas [see 26]. Our findings corroborate this insufficiency, indicating that there is a need for expanding the coverage of PAs to ensure the effective protection of angiosperm species. Furthermore, considering the rapid rate of habitat loss and increasing threats to biodiversity, it is crucial to prioritize the establishment of new PAs in areas with high angiosperm diversity and endemism. Expanding the coverage of protected areas to include these high diversity and endemism areas would help safeguard a larger proportion of angiosperm species.

Added to this, Amazonia stands out as having a significant knowledge gap; we are still far from knowing the likely species totals there. This vast, remote, and inaccessible area poses challenges for comprehensive botanical studies. A recent analysis that integrates ecological community metadata for multiple organism groups concluded that 15%–18% of the most neglected areas of Brazilian Amazonia will experience severe climate changes by 2050 and habitat destruction [27]. Therefore, additional efforts will be necessary in all Brazilian territory to surmount the challenges posed by regions characterized by limited accessibility and inadequate research infrastructure (mainly in Amazonia). There is a clear need for a national strategy for cataloging Amazonian plant species, funding for facilities for collection and research, and instruction for taxonomists working in the field. Also, for Caatinga, which in our study had the highest predicted proportion of undescribed species.

To address the challenges posed by inaccessible areas, collaboration between researchers, government agencies, and local communities is crucial. This can involve establishing partnerships with indigenous communities and investing in capacity building programs to train local individuals as botanists and taxonomists. Additionally, the use of innovative technologies such as remote sensing and drones can aid in the collection of data from these remote regions, facilitating more comprehensive botanical studies.

Alarmingly, recent data indicates that three out of four undescribed plant species (75%) are at risk of extinction [28]. This means that many species will be likely vanish before they can even be identified and studied. These projections of rapid biodiversity decline serve as a pressing motivation for intensifying our efforts to understand and document the species that still exist on Earth.

Indigenous lands, covering 115.3 million hectares, are the most preserved areas in Brazil, with only 1.2% native vegetation loss over 30 years. In comparison, private lands have lost 19.9% of their vegetation cover in the same period [65]. Based on these data, we infer that approximately 50% of the areas predicted to harbor the highest number of undescribed angiosperm species are relatively well protected. However, recent data highlights a significant growth in mining areas in the Amazon between 2020 and 2021, with Indigenous Lands featuring the largest mining areas and the highest number of landing strips—21 runways in the Xingu Park alone [65]. Additionally, fire has been a frequent issue on indigenous lands [66].

These pressures suggest that indigenous lands alone cannot fully protect native forests and undescribed angiosperms. On the other hand, our findings highlight the critical role of Indigenous lands in safeguarding areas with the highest potential for angiosperm discoveries in Brazil. Our analysis supports the hypothesis that undescribed species are concentrated in these areas, which underscores not only their biological richness, but also the effectiveness of traditional governance systems in preserving ecological integrity. This challenges prevailing conservation models that rely primarily on formally designated protected areas. Consistent with findings by Dawson et al. [67], who demonstrate that more equitable governance, especially when Indigenous peoples and local communities hold primary control, is associated with significantly more positive ecological outcomes, our results emphasize the importance of respecting and supporting customary institutions. These outcomes may reflect the active role of Indigenous peoples in managing and governing their territories, based on customary institutions that contribute to long-term ecological stewardship. Community-led monitoring and partnerships with Indigenous organizations can play a strategic role in improving botanical knowledge, while also reinforcing the protection of biologically rich and culturally significant landscapes. Integrating Indigenous lands more explicitly into national conservation planning could help accelerate both biodiversity discovery and protection. Policies that foster community-led research, strengthen biocultural knowledge systems, and reinforce Indigenous rights are essential to align scientific and local priorities and to ensure equitable, long-term conservation outcomes.

Achieving this requires closer collaboration between scientists, policymakers, and the public not only to raise awareness and support for taxonomic research, but also to promote effective protection for indigenous lands.

Bridging the gap between conservation science and policy implementation is crucial. By supporting indigenous communities, enforcing stricter regulations on activities like mining, and investing in research and conservation efforts, we can work towards a sustainable future that preserves both the ecological integrity of the Amazon and the rich diversity of its angiosperms. Only through collaborative efforts can we protect these invaluable resources for future generations.

## Conclusion

Biodiversity decline is among the most significant contemporary challenges driven by human activity. We are currently witnessing a biodiversity crisis, with the planet on the verge of the sixth mass extinction of plant diversity [68]. Despite the growing accessibility of global datasets, our understanding of biodiversity remains incomplete, particularly in the vast regions of the tropics that still lack comprehensive study. Fewer than 20% of living plant species have been assessed on the IUCN Red List of Threatened Species [69], even though an estimated 45% of them are currently under threat of extinction [70]. Although the number of plants documented to be at risk of extinction is greater than the sum of all the birds, amphibians, mammals, fish, and reptiles described [71], attention and funding devoted to plant conservation remain significantly lower than that devoted to animal conservation [31,32,68]. These numbers demonstrate the dire conservation situation of the world’s plants.

Despite the high levels of extinction risk for plant species [71], the actual number of threatened species may be underestimated, as it does not account for species that remain unknown to science. Undescribed species are predominantly range-restricted and/or rare, making them highly susceptible to extinction [28]. The increase in extinction risk over time [see 28] for newly described species suggests that rarity is a significant factor in their vulnerability. These species have restricted distributions because they are frequently hidden in areas with complex geological formations, varied climates, and intricate topography. Rare plant species often have reduced population genetic diversity, depressed fitness, limiting their capacity to adapt and increasing their susceptibility to rapid environmental changes. Consequently, their discovery becomes a challenging endeavor, rendering them even more vulnerable to extinction. Furthermore, with the rapid pace of human–induced climate change, species characterized by a high level of endemism might face challenges in adapting quickly enough to escape extinction [72].

One of the fundamental steps to preserving biodiversity is predicting future rates of species discovery and the number of unknown species [73]. Predicting how many species remain to be described and from where enables conservation resources to be scaled and allocated appropriately and conservation efforts to be better directed towards protecting threatened areas and species before they become endangered or extinct. The results of the present analysis, using botanical knowledge accumulated over centuries of description, filled a significant gap by conducting an original study aimed at accelerating the description of new taxa and providing the necessary link between taxonomy and conservation [see 74].

In our study, the dataset provided an accurate snapshot of the discovery potential and corroborated its effectiveness to detect knowledge gaps in the field and its greater potential for discovering new species.

This comprehensive analysis provides valuable insights into the species discovery potential, emphasizing the critical role of methodological approaches in biodiversity studies. The observed differences in species prediction between the basionym–inclusive and all–names models suggest that future research should carefully consider the historical context of species descriptions to enhance the accuracy of biodiversity estimates.

Our findings show an imbalance in knowledge of the Southeast compared to the North and Northeast of Brazil. The inventory of the southeast of Brazil is significantly more complete than the north and northeast of Brazil. Since almost 45% of the area of Brazilian Amazonia remains unsampled we are probably still far from accurate estimates of likely levels of diversity there. There is a clear need for a national strategy for cataloging angiosperm species, including funding for collection facilities, research, and training for taxonomists working in the field. In particular, the Amazon and Caatinga regions, which our study identified as having the highest proportion of undescribed species, will require significant efforts to overcome challenges related to limited accessibility and inadequate research infrastructure.

Although taxonomy plays an essential role in managing biodiversity information, it has been considered obscure, ambiguously defined, outdated, and lacking in prestige [4,5]. The reduction in investment in the area in recent years in Brazil [52,53], will make the process between the first collection of a putative new species and its description slower and more difficult, with a delay in publishing. This, in turn, can lead to delays in conservation efforts and hinder our understanding of biodiversity. Therefore, it is necessary to revalorise the science of taxonomy with renewed interest in exploration, more funding for the formation of new expertise, and more jobs for those who have already graduated. This can be achieved through collaborations between scientists, policymakers, and the public to increase awareness and support for taxonomy. It is also important to invest in technology and innovative methods to improve the efficiency and accuracy of taxonomic research.

The comprehensive documentation of the taxonomic catalog holds the potential for numerous benefits across various sectors, including agriculture, the pharmaceutical industry, and biodiversity [52]. By investing in taxonomic research, countries can gain a deeper understanding of their own ecosystems and make informed decisions regarding conservation efforts and resource management. Therefore, prioritizing taxonomic cataloging is not only crucial for scientific advancement but also has far–reaching implications for economic growth and environmental sustainability. Recognizing these advantages, Australia, for instance, has committed $824 million to enhance the state of taxonomic research and document the remaining species within the country [52,75]. With its vast diversity one might naturally assume that biodiversity and conservation research would be a priority in Brazil. However, despite an increase in research funding from 2000 to 2013 there has been a retrogression in science and education from 2019 to 2022 [52,53]. It is imperative for decision-makers change this perspective to genuinely recognize the importance of taxonomy and allocate the essential and deserved resources for this field. Brazil’s increased focus on developing its bioeconomy has included encouraging steps in this direction: the operationalization of Brazil’s National Fund for Access and Benefit-sharing is based on an admirably inclusive circumscription of potential beneficiaries which includes biodiversity-focused research institutions and collections https://www.planalto.gov.br/ccivil_03/_ato2023-2026/2024/decreto/D12044.htm.

Finally, Brazil harbors the greatest diversity of plant species on Earth [21], with at least ~7000–10000 undescribed species predicted in this analysis. Nevertheless, deforestation over the last thirty years has resulted in the immense loss of millions of hectares of natural habitats (see INPE http://terrabrasilis.dpi.inpe.br/app/dashboard/fires/biomes/aggregated/). The sustainable development of Brazil depends on balancing economic growth with conservation—a critical synergy for the preservation of species crucial to our planet. Understanding the potential locations of undescribed species is essential for informed decision–making regarding habitat protection and conservation management [19]. Thus, we hope that the consistent patterns of areas with high potential for species discovery identified in this study may serve as one of the criteria for defining new priority conservation areas in Brazil, reinforcing the urgency of implementing conservation measures to safeguard undescribed species, their unique attributes, and Brazilian centers of endemism from extinction.

## Supporting information

S1 Appendix Details of the statistical methods. (PDF)

S2 Appendix Reference of occurrence records by family. (PDF)

S3 Appendix Details of the taxonomic effort questionnaire by interview. (PDF)

S4 Appendix Temporal description of families of angiosperms species. The species accumulation curve, and the fit of the nonlinear models to the data. According to Prediction Model Results: Gompertz 3P, Gompertz 4P, Logistic 3P, and Weibull Growth models for the top 10 angiosperm families with the highest potential for species discoveries.
(PDF)

S5 Appendix Table with parameter estimates and maximum–likelihood confidence intervals (CIs), Akaike’s Information Criterion (AIC), AICc weight, and Bayesian Information Criterion (BIC) according to prediction model results: Gompertz 3P, Gompertz 4P, Logistic 3P, and Weibull Growth models for the top 10 angiosperm families with the highest potential for species discoveries. (PDF)

S6 Appendix Complete table with all models and differences (when found) executed in R and JMP. Comparison of models, number of known and predicted species, and percentage of expected increase, of flowering plants, by the Brazilian phytogeographic domains. For cases where the Weibull Growth model did not converge to real values (BIC number, AIC weight, or asymptote value), these are indicated by a ‘–’ in the table. (R) and (JMP) next to the model name indicate where the data was generated. Models without specifications indicate no differences in results between the execution programs. Models with the best fit for the evaluated database are highlighted in bold.
(PDF)

S7 Appendix Cumulative historical deforestation data up to 2022, retrieved from the Amazon Deforestation Estimation Project (PRODES). (PDF)

S1 Fig Completeness of the taxonomic inventory by polygon at each resolution (a-f), density analysis (g), and a comprehensive map showing the proportion of future discoveries for Brazilian angiosperms (h). (PDF)
