# Supplementary material for: Protecting hidden treasures: Indigenous lands safeguard 50% of areas with the highest potential for angiosperm discoveries in Brazil—patterns and conservation priorities
Source: PLoS One. 2025 Jul 9;20(7):e0326507. doi: 10.1371/journal.pone.0326507 (PMC12240397; doi:10.1371/journal.pone.0326507)
Supplement: S1 Appendix — (PDF) [file pone.0326507.s001.pdf]

Protecting Hidden Treasures: Indigenous Lands Safeguard 50% of Areas with the Highest Potential for Angiosperm Discoveries in Brazil – Patterns and Conservation Priorities

Janaína Gomes-da-Silva[1,*]
Eimear Nic Lughadha[2]
Rafaela Campostrini Forzza[1,3]

[1]Jardim Botânico do Rio de Janeiro, Rua Pacheco Leão, 915, Rio de Janeiro, RJ, 2460–030, Brazil.
[2]Science Directorate, Royal Botanic Gardens, Kew, Richmond, TW9 3AE, UK
[3]Instituto Chico Mendes de Conservação da Biodiversidade, Parque Nacional do Descobrimento, Bahia, Brazil.
[*] Author for Correspondence: jgomes_da_silva@yahoo.com.br

Supporting Information

Appendix S1. Details of the statistical methods.

Growth rate per unit of time or discovery rate

Growth rate = (Final count - Initial count) / Time

Prediction Model
Gompertz 3P

$$a \cdot \mathrm{Exp}\left(-\mathrm{Exp}\left(-b \cdot \left(\mathrm{year} - c\right)\right)\right)$$

f(year) = Asym * exp(-exp(scal * (year - xmid)))

The basic syntax of the function is as follows:
a = Asymptote
b = Growth Rate
c = Inflection Point
f(year) = Asym * exp(-exp(scal * (year - xmid)))

Gompertz 4P
Prediction Model

$$a + \left(b - a\right) \cdot \mathrm{Exp}\left(-\mathrm{Exp}\left(-c \cdot \left(\mathrm{Column\ 1} - d\right)\right)\right)$$

a = Lower Asymptote
b = Upper Asymptote
c = Growth Rate
d = Inflection Point

Logistic 3P
Prediction Model

$$\frac{c}{\left(1+\mathrm{Exp}\left(-a\cdot\left(\mathrm{year}-b\right)\right)\right)}$$

a = Growth Rate
b = Inflection Point
c = Asymptote

Weibull Growth
Prediction Model

$$a\cdot\left(1-\mathrm{Exp}\left(-\left(\frac{\mathrm{year}}{b}\right)^{c}\right)\right)$$

a = Asymptote
b = Inflection Point
c = Growth Rate

The Gompertz model is a mathematical model that describes the initial exponential growth of a system, followed by slower growth until reaching an asymptote. This model is defined by an equation with two parameters: the asymptote (the maximum value the system can reach) and the growth rate.

On the other hand, the 3-parameter Gompertz model is an extension of the Gompertz model, adding a third parameter that represents the horizontal displacement (or lag) of the start of growth relative to time. This parameter allows for the adjustment of cases where growth does not begin immediately from zero.

Gompertz 4P Model: The 4-parameter Gompertz model is an extension of the traditional Gompertz model that includes an additional parameter to further refine the model's fit to data. The four parameters in the Gompertz 4P model are: asymptote (A): The maximum value that the system can reach; Growth Rate (B): The rate at which the system grows;

Horizontal Displacement (C): The time lag before the growth starts; Vertical Displacement (D): A parameter that shifts the curve vertically to better fit data points that do not start from zero. This additional vertical displacement parameter (D) allows the Gompertz 4P model to more accurately represent datasets where the initial value is not zero, providing a more flexible and precise fit.

Logistic 3P Model: The 3-parameter logistic model describes population growth that starts exponentially, then slows as the population approaches the carrying capacity of the environment. This model is defined by three parameters: the carrying capacity (the maximum population size that the environment can sustain), the growth rate, and the inflection point (the point at which the growth rate is highest).

Weibull Growth Model: The Weibull growth model is used to describe growth processes that may not follow a simple exponential or logistic pattern. It is particularly useful for modeling systems where the growth rate changes non-linearly over time. The Weibull model is characterized by its flexibility, allowing it to fit a wide range of data patterns by adjusting its shape parameters.

Selection Method: To select the best model, we fitted each model to the data and compared the fit quality using criteria such as AIC (Akaike Information Criterion), Akaike weight (a model whose Akaike weight approaches 1 is unambiguously supported by the data), and BIC (Bayesian Information Criterion—lower BIC values indicate a better model). These criteria help identify the model that best balances data fit and model complexity. AIC and BIC are robust approaches as they penalize model complexity, helping to avoid overfitting.

BIC: The BIC is useful when one is more interested in avoiding overfitting, meaning when one wants to penalize more complex models. It tends to select simpler models and is therefore preferred when there is a greater concern for the generalization of the model to new data (Bayesian Information Criterion—lower BIC values indicate a better model).

AIC: The AIC is useful when one is more interested in finding the model that best describes the observed data, even if it means a slightly more complex model. It tends to select models

that fit the observed data well and is preferred when one wants to understand the underlying processes that generate the data (Akaike Information Criterion—models with lower AIC are preferred).

AICc Weight: The AICc weight is useful when one wants to have an idea of the relative probability of each model being the true data-generating model, considering both the model fit and the penalization for complexity. It provides a measure of uncertainty about the best model choice and is preferred when one wants a more probabilistic approach to model selection (Akaike weight—a model whose Akaike weight approaches 1 is unambiguously supported by the data).

Both AIC and BIC criteria help balance model complexity and fit to the data. However, BIC imposes a greater penalty for complexity, making it more effective in preventing overfitting compared to AIC. Hypotheses with an AIC Weight closer to 1 are preferred. When there is more than one hypothesis with values close to 1, as in the case of the Pampa, models with lower BIC and AIC values are chosen to determine the best hypothesis.

Completeness index

Completeness Index = (Observed Species Richness / Estimated Total Species Richness) *100

Statistical estimate of the total number of species

For each ten-year interval, we calculate the number of unique species discovered and the number of taxonomists working.

The remaining number of species to be described:

$S_R = S_T - \Sigma Si$

SR= It is the total number of species, ST, – which is to be estimated – minus the cumulative number of species already described, ΣSi up to the given year, (Joppa et al. 2011).

$Si = (a + b\ Y_i) * (T_i) * (S_T - \Sigma Si)$

Si is the number of species described per unit time, Yi the time interval, Ti the number of taxonomists involved in the description, and ΣSi the total number of species described to

that time; a, b, and ST are constants to be estimated, the last one being the predicted total number of species (Joppa et al. 2011).
