# Supplementary material for: Protecting hidden treasures: Indigenous lands safeguard 50% of areas with the highest potential for angiosperm discoveries in Brazil—patterns and conservation priorities
Source: PLoS One. 2025 Jul 9;20(7):e0326507. doi: 10.1371/journal.pone.0326507 (PMC12240397; doi:10.1371/journal.pone.0326507)
Supplement: S2 Appendix — (PDF) [file pone.0326507.s002.pdf]

Protecting Hidden Treasures: Indigenous Lands Safeguard 50% of Areas with the Highest Potential for Angiosperm Discoveries in Brazil – Patterns and Conservation Priorities

Janaína Gomes-da-Silva[1,*]
Eimear Nic Lughadha[2]
Rafaela Campostrini Forzza[1,3]

[1]Jardim Botânico do Rio de Janeiro, Rua Pacheco Leão, 915, Rio de Janeiro, RJ, 2460–030, Brazil.
[2]Science Directorate, Royal Botanic Gardens, Kew, Richmond, TW9 3AE, UK
[3]Instituto Chico Mendes de Conservação da Biodiversidade, Parque Nacional do Descobrimento, Bahia, Brazil.
[*] Author for Correspondence: jgomes_da_silva@yahoo.com.br

Supporting Information

Appendix S2. Reference of occurrence records by family.
GBIF

GBIF.org (13 March 2023) GBIF Occurrence Download

Acanthaceae GBIF.org (14 March 2023) GBIF Occurrence Download

https://doi.org/10.15468/dl.evds78

Achariaceae

GBIF.org (14 March 2023) GBIF Occurrence Download

https://doi.org/10.15468/dl.4za5h6

Achatocarpaceae

GBIF.org (14 March 2023) GBIF Occurrence Download

https://doi.org/10.15468/dl.n3vbwm

Actinidiaceae

GBIF.org (14 March 2023) GBIF Occurrence Download

https://doi.org/10.15468/dl.g3wjqx

Adoxaceae

Aizoaceae

GBIF.org (14 March 2023) GBIF Occurrence Download

https://doi.org/10.15468/dl.9953x5

Alismataceae

GBIF.org (15 March 2023) GBIF Occurrence Download

https://doi.org/10.15468/dl.ecjbzu

Alstroemeriaceae

GBIF.org (15 March 2023) GBIF Occurrence Download

https://doi.org/10.15468/dl.t3chx3

Amaranthaceae

GBIF.org (15 March 2023) GBIF Occurrence Download

https://doi.org/10.15468/dl.dz4pdc

Amaryllidaceae

https://api.gbif.org/v1/occurrence/download/request/0094069-230224095556074.zip

Anacardiaceae

GBIF.org (15 March 2023) GBIF Occurrence Download

https://doi.org/10.15468/dl.xeqej5

Anisophylleaceae

GBIF.org (15 March 2023) GBIF Occurrence Download

https://doi.org/10.15468/dl.aycesd

Annonaceae

GBIF.org (15 March 2023) GBIF Occurrence Download

https://doi.org/10.15468/dl.2th3fj

Apiaceae

GBIF.org (15 March 2023) GBIF Occurrence Download

https://doi.org/10.15468/dl.9g4umy

Apocynaceae

GBIF.org (16 March 2023) GBIF Occurrence Download

https://doi.org/10.15468/dl.jtaztb

Apodanthaceae

https://api.gbif.org/v1/occurrence/download/request/0095755-230224095556074.zip

Aptandraceae

https://api.gbif.org/v1/occurrence/download/request/0095781-230224095556074.zip

Aquifoliaceae

GBIF.org (16 March 2023) GBIF Occurrence Download

https://doi.org/10.15468/dl.db2vvm

Araceae

GBIF.org (16 March 2023) GBIF Occurrence Download

https://doi.org/10.15468/dl.y3xrzh

Araliaceae

GBIF.org (16 March 2023) GBIF Occurrence Download

https://doi.org/10.15468/dl.ujfsyf

Arecaceae

GBIF.org (16 March 2023) GBIF Occurrence Download

https://doi.org/10.15468/dl.sh58wb

Aristolochiaceae

GBIF.org (16 March 2023) GBIF Occurrence Download

https://doi.org/10.15468/dl.keyczn

Asparagaceae

GBIF.org (16 March 2023) GBIF Occurrence Download

https://doi.org/10.15468/dl.wafqqh

Asteraceae

GBIF.org (16 March 2023) GBIF Occurrence Download

https://doi.org/10.15468/dl.3uhmu7

Balanophoraceae

GBIF.org (16 March 2023) GBIF Occurrence Download

https://doi.org/10.15468/dl.phggvg

Basellaceae

GBIF.org (16 March 2023) GBIF Occurrence Download

https://doi.org/10.15468/dl.wjamcj

Begoniaceae

GBIF.org (16 March 2023) GBIF Occurrence Download

https://doi.org/10.15468/dl.jxuwmb

Berberidaceae

GBIF.org (16 March 2023) GBIF Occurrence Download

https://doi.org/10.15468/dl.mzneqt

Bignoniaceae

GBIF.org (16 March 2023) GBIF Occurrence Download

https://doi.org/10.15468/dl.y45kfu

Bixaceae

GBIF.org (16 March 2023) GBIF Occurrence Download

https://doi.org/10.15468/dl.7gygx8

Bonnetiaceae

GBIF.org (16 March 2023) GBIF Occurrence

Download https://doi.org/10.15468/dl.xuyr36

Boraginaceae

GBIF.org (16 March 2023) GBIF Occurrence Download

https://doi.org/10.15468/dl.7vf2yt

Brassicaceae

GBIF.org (16 March 2023) GBIF Occurrence Download

https://doi.org/10.15468/dl.ngv7jc

Bromeliaceae

GBIF.org (16 March 2023) GBIF Occurrence Download

https://doi.org/10.15468/dl.2qgdm4

Brunelliaceae

GBIF.org (16 March 2023) GBIF Occurrence Download

https://doi.org/10.15468/dl.7wvgkb

Burmanniaceae

GBIF.org (16 March 2023) GBIF Occurrence Download

https://doi.org/10.15468/dl.r6b3ng

Burseraceae

GBIF.org (16 March 2023) GBIF Occurrence Download

https://doi.org/10.15468/dl.chugdr

Cabombaceae

GBIF.org (16 March 2023) GBIF Occurrence Download

https://doi.org/10.15468/dl.sxeqwu

Cactaceae

GBIF.org (17 March 2023) GBIF Occurrence Download

https://doi.org/10.15468/dl.sj3xme

Calophyllaceae

GBIF.org (17 March 2023) GBIF Occurrence Download

https://doi.org/10.15468/dl.9xpemp

Calyceraceae

GBIF.org (17 March 2023) GBIF Occurrence Download

https://doi.org/10.15468/dl.2gst6w

Campanulaceae

GBIF.org (17 March 2023) GBIF Occurrence Download

https://doi.org/10.15468/dl.q9q4w7

Canellaceae

GBIF.org (17 March 2023) GBIF Occurrence Download

https://doi.org/10.15468/dl.s6x5yv

Cannabaceae

https://api.gbif.org/v1/occurrence/download/request/0100130-230224095556074.zip

Cannaceae

https://api.gbif.org/v1/occurrence/download/request/0100137-230224095556074.zip

Capparaceae

GBIF.org (17 March 2023) GBIF Occurrence Download https://doi.org/10.15468/dl.aqdvxf

Caprifoliaceae

GBIF.org (17 March 2023) GBIF Occurrence Download https://doi.org/10.15468/dl.8uxzc7

Cardiopteridaceae

GBIF.org (17 March 2023) GBIF Occurrence Download https://doi.org/10.15468/dl.5mtgjn

Caricaceae

GBIF.org (19 March 2023) GBIF Occurrence Download https://doi.org/10.15468/dl.bh25uf

Caryocaraceae

GBIF.org (17 March 2023) GBIF Occurrence Download

https://doi.org/10.15468/dl.avnzg7

Caryophyllaceae

GBIF.org (17 March 2023) GBIF Occurrence Download

https://doi.org/10.15468/dl.6258sy

Celastraceae

GBIF.org (17 March 2023) GBIF Occurrence Download

https://doi.org/10.15468/dl.ec2suu

Ceratophyllaceae

GBIF.org (17 March 2023) GBIF Occurrence Download

https://doi.org/10.15468/dl.kb46vb

Chloranthaceae

GBIF.org (17 March 2023) GBIF Occurrence Download

https://doi.org/10.15468/dl.f7rdhn

Chrysobalanaceae

GBIF.org (17 March 2023) GBIF Occurrence Download

https://doi.org/10.15468/dl.qjjsqw

Cistaceae

GBIF.org (17 March 2023) GBIF Occurrence Download

https://doi.org/10.15468/dl.nxnbz6

Cleomaceae

GBIF.org (17 March 2023) GBIF Occurrence Download

https://doi.org/10.15468/dl.bx4gn4

Clethraceae

GBIF.org (17 March 2023) GBIF Occurrence Download

https://doi.org/10.15468/dl.cmvmw6

Clusiaceae

GBIF.org (17 March 2023) GBIF Occurrence Download

https://doi.org/10.15468/dl.s33r84

Combretaceae

GBIF.org (17 March 2023) GBIF Occurrence Download

https://doi.org/10.15468/dl.sfutrw

Commelinaceae

GBIF.org (17 March 2023) GBIF Occurrence Download

https://doi.org/10.15468/dl.8fnbqh

Connaraceae

GBIF.org (17 March 2023) GBIF Occurrence Download

https://doi.org/10.15468/dl.syrwkx

Convolvulaceae

GBIF.org (17 March 2023) GBIF Occurrence Download

https://doi.org/10.15468/dl.p8j2fz

Costaceae

GBIF.org (17 March 2023) GBIF Occurrence Download

https://doi.org/10.15468/dl.txzcar

Coulaceae

GBIF.org (17 March 2023) GBIF Occurrence Download

https://doi.org/10.15468/dl.ts5vg5

Crassulaceae

GBIF.org (17 March 2023) GBIF Occurrence Download

https://doi.org/10.15468/dl.7njjyc

Cucurbitaceae

GBIF.org (17 March 2023) GBIF Occurrence Download

https://doi.org/10.15468/dl.tarf6f

Cunoniaceae

GBIF.org (17 March 2023) GBIF Occurrence Download

https://doi.org/10.15468/dl.6grwn3

Cyclanthaceae

GBIF.org (17 March 2023) GBIF Occurrence Download

https://doi.org/10.15468/dl.sbm525

Cymodoceaceae

GBIF.org (17 March 2023) GBIF Occurrence Download

https://doi.org/10.15468/dl.e5ghew

Cyperaceae

GBIF.org (17 March 2023) GBIF Occurrence Download

https://doi.org/10.15468/dl.btftk6

Dichapetalaceae

GBIF.org (20 March 2023) GBIF Occurrence Download

https://doi.org/10.15468/dl.gqsa62

Dilleniaceae

GBIF.org (20 March 2023) GBIF Occurrence Download

https://doi.org/10.15468/dl.gq8tmh

Dioscoreaceae

https://api.gbif.org/v1/occurrence/download/request/0104214-230224095556074.zip

Droseraceae

GBIF.org (20 March 2023) GBIF Occurrence Download

https://doi.org/10.15468/dl.h4d29j

Ebenaceae

GBIF.org (20 March 2023) GBIF Occurrence Download

https://doi.org/10.15468/dl.jbbv44

Elaeocarpaceae

GBIF.org (20 March 2023) GBIF Occurrence Download

https://doi.org/10.15468/dl.ky37gu

Ehretiaceae

GBIF.org (23 April 2023) GBIF Occurrence Download

https://doi.org/10.15468/dl.u65h2r

Elatinaceae

GBIF.org (20 March 2023) GBIF Occurrence Download

https://doi.org/10.15468/dl.5fzkr5

Ericaceae

GBIF.org (20 March 2023) GBIF Occurrence Download

https://doi.org/10.15468/dl.x49cdm

Eriocaulaceae

GBIF.org (20 March 2023) GBIF Occurrence Download

https://doi.org/10.15468/dl.p62jc2

Erythropalaceae

GBIF.org (20 March 2023) GBIF Occurrence Download

https://doi.org/10.15468/dl.vuxxsb

Erythroxylaceae

GBIF.org (20 March 2023) GBIF Occurrence Download

https://doi.org/10.15468/dl.cqj3qb

Escalloniaceae

GBIF.org (20 March 2023) GBIF Occurrence Download

https://doi.org/10.15468/dl.84vsaa

Euphorbiaceae

GBIF.org (20 March 2023) GBIF Occurrence Download

https://doi.org/10.15468/dl.3xvh5t

Euphroniaceae

GBIF.org (20 March 2023) GBIF Occurrence Download

https://doi.org/10.15468/dl.wsxbut

Fabaceae

GBIF.org (20 March 2023) GBIF Occurrence Download

https://doi.org/10.15468/dl.fsb6qg

Gelsemiaceae

GBIF.org (20 March 2023) GBIF Occurrence Download

https://doi.org/10.15468/dl.g4x5sa

Gentianaceae

GBIF.org (20 March 2023) GBIF Occurrence Download

https://doi.org/10.15468/dl.vg7kje

Geraniaceae

GBIF.org (20 March 2023) GBIF Occurrence Download

https://doi.org/10.15468/dl.u9jaat

Gesneriaceae

GBIF.org (20 March 2023) GBIF Occurrence Download

https://doi.org/10.15468/dl.4e7cad

Goodeniaceae

Goupiaceae

GBIF.org (20 March 2023) GBIF Occurrence Download

https://doi.org/10.15468/dl.h4jnh8

Griseliniaceae

GBIF.org (20 March 2023) GBIF Occurrence Download

https://doi.org/10.15468/dl.3vk5fh

Gunneraceae

GBIF.org (20 March 2023) GBIF Occurrence Download

https://doi.org/10.15468/dl.4wmt33

Haemodoraceae

GBIF.org (20 March 2023) GBIF Occurrence Download

https://doi.org/10.15468/dl.rkx6h7

Haloragaceae

GBIF.org (20 March 2023) GBIF Occurrence Download

https://doi.org/10.15468/dl.6qn24n

Heliconiaceae

GBIF.org (20 March 2023) GBIF Occurrence Download

https://doi.org/10.15468/dl.wrf9xu

Hernandiaceae

GBIF.org (20 March 2023) GBIF Occurrence Download

https://doi.org/10.15468/dl.pqbn8w

Humiriaceae

GBIF.org (20 March 2023) GBIF Occurrence Download

https://doi.org/10.15468/dl.7mub5t

Hydnoraceae

GBIF.org (20 March 2023) GBIF Occurrence Download

https://doi.org/10.15468/dl.der86h

Hypericaceae

GBIF.org (20 March 2023) GBIF Occurrence Download

https://doi.org/10.15468/dl.jyt6m2

Hydrocharitaceae

GBIF.org (20 March 2023) GBIF Occurrence Download

https://doi.org/10.15468/dl.cy5m83

Hydroleaceae

Hypoxidaceae

GBIF.org (20 March 2023) GBIF Occurrence Download

https://doi.org/10.15468/dl.dtmqb8

Icacinaceae

GBIF.org (20 March 2023) GBIF Occurrence Download

https://doi.org/10.15468/dl.5uc4pp

Iridaceae

GBIF.org (20 March 2023) GBIF Occurrence Download

https://doi.org/10.15468/dl.wfxrd7

Ixonanthaceae

GBIF.org (20 March 2023) GBIF Occurrence Download

https://doi.org/10.15468/dl.eh2tnw

Juglandaceae

GBIF.org (21 March 2023) GBIF Occurrence Download

https://doi.org/10.15468/dl.7gz7xv

Juncaceae

GBIF.org (21 March 2023) GBIF Occurrence Download

https://doi.org/10.15468/dl.2jtbqr

Juncaginaceae

GBIF.org (21 March 2023) GBIF Occurrence Download

https://doi.org/10.15468/dl.m8ubw5

Krameriaceae

GBIF.org (21 March 2023) GBIF Occurrence Download

https://doi.org/10.15468/dl.hmeh8m

Lacistemataceae

GBIF.org (21 March 2023) GBIF Occurrence Download

https://doi.org/10.15468/dl.3vw45c

Lamiaceae

GBIF.org (21 March 2023) GBIF Occurrence Download

https://doi.org/10.15468/dl.aa4qc4

Lauraceae

GBIF.org (21 March 2023) GBIF Occurrence Download

https://doi.org/10.15468/dl.k64r9a

Lecythidaceae

GBIF.org (21 March 2023) GBIF Occurrence Download

https://doi.org/10.15468/dl.3xamcn

Lentibulariaceae

GBIF.org (21 March 2023) GBIF Occurrence Download

https://doi.org/10.15468/dl.avzyby

Lepidobotryaceae

Linaceae

GBIF.org (21 March 2023) GBIF Occurrence Download

https://doi.org/10.15468/dl.q3ctxs

Linderniaceae

GBIF.org (21 March 2023) GBIF Occurrence Download

https://doi.org/10.15468/dl.wbuhaf

Loasaceae

GBIF.org (21 March 2023) GBIF Occurrence Download

https://doi.org/10.15468/dl.wmfjfz

Loganiaceae

GBIF.org (21 March 2023) GBIF Occurrence Download

https://doi.org/10.15468/dl.zhgcsm

Loranthaceae

GBIF.org (21 March 2023) GBIF Occurrence Download

https://doi.org/10.15468/dl.cmrfem

Lythraceae

GBIF.org (21 March 2023) GBIF Occurrence Download

https://doi.org/10.15468/dl.xrgpce

Magnoliaceae

GBIF.org (21 March 2023) GBIF Occurrence Download

https://doi.org/10.15468/dl.gg8har

Malpighiaceae

GBIF.org (21 March 2023) GBIF Occurrence Download

https://doi.org/10.15468/dl.6rzc52

Malvaceae

GBIF.org (21 March 2023) GBIF Occurrence Download

https://doi.org/10.15468/dl.9atsv5

Marantaceae

GBIF.org (21 March 2023) GBIF Occurrence

Download https://doi.org/10.15468/dl.wxyu9m

Marcgraviaceae

GBIF.org (21 March 2023) GBIF Occurrence Download

https://doi.org/10.15468/dl.sm5stt

Martyniaceae

GBIF.org (21 March 2023) GBIF Occurrence Download

https://doi.org/10.15468/dl.5ynpyz

Mayacaceae

GBIF.org (21 March 2023) GBIF Occurrence Download

https://doi.org/10.15468/dl.a4aycn

Melastomataceae

GBIF.org (21 March 2023) GBIF Occurrence Download

https://doi.org/10.15468/dl.26vruf

Meliaceae

GBIF.org (21 March 2023) GBIF Occurrence Download

https://doi.org/10.15468/dl.qpwyg8

Menispermaceae

GBIF.org (21 March 2023) GBIF Occurrence Download

https://doi.org/10.15468/dl.tjbfch

Menyanthaceae

Metteniusaceae

GBIF.org (21 March 2023) GBIF Occurrence Download

https://doi.org/10.15468/dl.zwpxhf

Microteaceae

GBIF.org (21 March 2023) GBIF Occurrence Download

https://doi.org/10.15468/dl.n7pgsk

Molluginaceae

GBIF.org (21 March 2023) GBIF Occurrence Download

https://doi.org/10.15468/dl.s7hdae

Monimiaceae

GBIF.org (21 March 2023) GBIF Occurrence Download

https://doi.org/10.15468/dl.ssssss

Moraceae

GBIF.org (21 March 2023) GBIF Occurrence Download

https://doi.org/10.15468/dl.macae3

Myristicaceae

GBIF.org (21 March 2023) GBIF Occurrence Download

https://doi.org/10.15468/dl.54g84h

Myrtaceae

GBIF.org (21 March 2023) GBIF Occurrence Download

https://doi.org/10.15468/dl.razh7x

Nyctaginaceae

GBIF.org (22 March 2023) GBIF Occurrence Download

https://doi.org/10.15468/dl.4zk88r

Nymphaeaceae

GBIF.org (22 March 2023) GBIF Occurrence Download

https://doi.org/10.15468/dl.mmwa8y

Ochnaceae

GBIF.org (22 March 2023) GBIF Occurrence Download

https://doi.org/10.15468/dl.fpegcf

Olacaceae

GBIF.org (22 March 2023) GBIF Occurrence Download

https://doi.org/10.15468/dl.ddgncc

Oleaceae

GBIF.org (22 March 2023) GBIF Occurrence Download

https://doi.org/10.15468/dl.vtcmf4

Onagraceae

GBIF.org (22 March 2023) GBIF Occurrence Download

https://doi.org/10.15468/dl.4vub2h

Opiliaceae

GBIF.org (22 March 2023) GBIF Occurrence Download

https://doi.org/10.15468/dl.8h65qs

Orchidaceae

GBIF.org (22 March 2023) GBIF Occurrence Download

https://doi.org/10.15468/dl.zfsgfv

Orobanchaceae

GBIF.org (22 March 2023) GBIF Occurrence Download

https://doi.org/10.15468/dl.q4as7h

Oxalidaceae

GBIF.org (22 March 2023) GBIF Occurrence Download

https://doi.org/10.15468/dl.h5zrg9

Passifloraceae

GBIF.org (22 March 2023) GBIF Occurrence Download

https://doi.org/10.15468/dl.2v4wgj

Pentaphylacaceae

GBIF.org (22 March 2023) GBIF Occurrence Download

https://doi.org/10.15468/dl.tdnsdb

Peraceae

GBIF.org (22 March 2023) GBIF Occurrence Download

https://doi.org/10.15468/dl.xcvgq7

Peridiscaceae

GBIF.org (22 March 2023) GBIF Occurrence Download

https://doi.org/10.15468/dl.6ksdy9

Phyllanthaceae

GBIF.org (22 March 2023) GBIF Occurrence Download

https://doi.org/10.15468/dl.tuqq32

Phytolaccaceae

GBIF.org (22 March 2023) GBIF Occurrence Download

https://doi.org/10.15468/dl.jkt47x

Picramniaceae

GBIF.org (22 March 2023) GBIF Occurrence Download

https://doi.org/10.15468/dl.7pkcnb

Picrodendraceae

GBIF.org (22 March 2023) GBIF Occurrence Download

https://doi.org/10.15468/dl.ye4rjp

Piperaceae

GBIF.org (22 March 2023) GBIF Occurrence Download

https://doi.org/10.15468/dl.66zrbp

Plantaginaceae

GBIF.org (22 March 2023) GBIF Occurrence Download

https://doi.org/10.15468/dl.rmwx7s

Plumbaginaceae

GBIF.org (22 March 2023) GBIF Occurrence Download

https://doi.org/10.15468/dl.smbggb

Poaceae

GBIF.org (22 March 2023) GBIF Occurrence Download

https://doi.org/10.15468/dl.tzzgmv

Podostemaceae

GBIF.org (22 March 2023) GBIF Occurrence Download

https://doi.org/10.15468/dl.x4s7mr

Polygalaceae

GBIF.org (22 March 2023) GBIF Occurrence Download

https://doi.org/10.15468/dl.82rbym

Polygonaceae

GBIF.org (22 March 2023) GBIF Occurrence Download

https://doi.org/10.15468/dl.6u6rjh

Pontederiaceae

GBIF.org (22 March 2023) GBIF Occurrence Download

https://doi.org/10.15468/dl.d9zcfp

Portulacaceae

GBIF.org (22 March 2023) GBIF Occurrence Download

https://doi.org/10.15468/dl.jp3nca

Potamogetonaceae

GBIF.org (22 March 2023) GBIF Occurrence Download

https://doi.org/10.15468/dl.zwchvm

Primulaceae

GBIF.org (22 March 2023) GBIF Occurrence Download

https://doi.org/10.15468/dl.c6sez4

Proteaceae

GBIF.org (22 March 2023) GBIF Occurrence Download

https://doi.org/10.15468/dl.akdcjr

Putranjivaceae

GBIF.org (22 March 2023) GBIF Occurrence Download

https://doi.org/10.15468/dl.n2pnnq

Quiinaceae

GBIF.org (22 March 2023) GBIF Occurrence Download

https://doi.org/10.15468/dl.s5myn9

Quillajaceae

GBIF.org (22 March 2023) GBIF Occurrence Download

https://doi.org/10.15468/dl.4bcfzd

Ranunculaceae

GBIF.org (22 March 2023) GBIF Occurrence Download

https://doi.org/10.15468/dl.w5apft

Rapateaceae

GBIF.org (22 March 2023) GBIF Occurrence Download

https://doi.org/10.15468/dl.jwzzdv

Rhabdodendraceae

GBIF.org (22 March 2023) GBIF Occurrence Download

https://doi.org/10.15468/dl.c9faem

Rhamnaceae

GBIF.org (22 March 2023) GBIF Occurrence Download

https://doi.org/10.15468/dl.jf6txt

Rhizophoraceae

GBIF.org (22 March 2023) GBIF Occurrence Download

https://doi.org/10.15468/dl.54pnkd

Rosaceae

GBIF.org (22 March 2023) GBIF Occurrence Download

https://doi.org/10.15468/dl.4cqvx8

Rubiaceae

GBIF.org (22 March 2023) GBIF Occurrence Download

https://doi.org/10.15468/dl.wxfv7x

Ruppiaceae

GBIF.org (22 March 2023) GBIF Occurrence Download

https://doi.org/10.15468/dl.4bj836

Rutaceae

GBIF.org (22 March 2023) GBIF Occurrence Download

https://doi.org/10.15468/dl.wxe5cn

Sabiaceae

GBIF.org (22 March 2023) GBIF Occurrence Download

https://doi.org/10.15468/dl.2cbvbx

Salicaceae

GBIF.org (22 March 2023) GBIF Occurrence Download

https://doi.org/10.15468/dl.rcmb6q

Santalaceae

GBIF.org (22 March 2023) GBIF Occurrence Download

https://doi.org/10.15468/dl.6at98f

Sapindaceae

GBIF.org (22 March 2023) GBIF Occurrence Download

https://doi.org/10.15468/dl.fuzkpp

Sapotaceae

GBIF.org (22 March 2023) GBIF Occurrence Download

https://doi.org/10.15468/dl.u5a586

Sarraceniaceae

GBIF.org (22 March 2023) GBIF Occurrence Download

https://doi.org/10.15468/dl.5cmhav

Schlegeliaceae

GBIF.org (22 March 2023) GBIF Occurrence Download

https://doi.org/10.15468/dl.fnm8ze

Schoepfiaceae

GBIF.org (22 March 2023) GBIF Occurrence Download

https://doi.org/10.15468/dl.86vk29

Scrophulariaceae

GBIF.org (22 March 2023) GBIF Occurrence Download

https://doi.org/10.15468/dl.5d75eq

Simaroubaceae

GBIF.org (22 March 2023) GBIF Occurrence Download

https://doi.org/10.15468/dl.fzbz3b

Siparunaceae

GBIF.org (22 March 2023) GBIF Occurrence Download

https://doi.org/10.15468/dl.7y4k5n

Smilacaceae

GBIF.org (22 March 2023) GBIF Occurrence Download

https://doi.org/10.15468/dl.2amfcq

Solanaceae

GBIF.org (22 March 2023) GBIF Occurrence Download

https://doi.org/10.15468/dl.du6x69

Stemonuraceae

GBIF.org (22 March 2023) GBIF Occurrence Download

https://doi.org/10.15468/dl.v5crmx

Strelitziaceae

GBIF.org (22 March 2023) GBIF Occurrence Download

https://doi.org/10.15468/dl.4xmcw9

Strombosiaceae

GBIF.org (22 March 2023) GBIF Occurrence Download

https://doi.org/10.15468/dl.tdtz6h

Styracaceae

GBIF.org (22 March 2023) GBIF Occurrence Download https://

doi.org/10.15468/dl.52v2wz

Symplocaceae

GBIF.org (22 March 2023) GBIF Occurrence Download

https://doi.org/10.15468/dl.ysha54

Talinaceae

GBIF.org (23 March 2023) GBIF Occurrence Download

https://doi.org/10.15468/dl.nmvqdg

Theaceae

GBIF.org (23 March 2023) GBIF Occurrence Download

https://doi.org/10.15468/dl.6cybt4

Thismiaceae

GBIF.org (23 March 2023) GBIF Occurrence Download

https://doi.org/10.15468/dl.h2uxfv

Thymelaeaceae

GBIF.org (23 March 2023) GBIF Occurrence Download

https://doi.org/10.15468/dl.k2pzjj

Trigoniaceae

GBIF.org (23 March 2023) GBIF Occurrence Download

https://doi.org/10.15468/dl.xkxbef

Triuridaceae

GBIF.org (23 March 2023) GBIF Occurrence Download

https://doi.org/10.15468/dl.8mhp7t

Tropaeolaceae

GBIF.org (23 March 2023) GBIF Occurrence Download

https://doi.org/10.15468/dl.m2ddxr

Turneraceae

GBIF.org (23 March 2023) GBIF Occurrence Download

https://doi.org/10.15468/dl.yutbft

Typhaceae

GBIF.org (23 March 2023) GBIF Occurrence Download

https://doi.org/10.15468/dl.jf8k4v

Ulmaceae

GBIF.org (23 March 2023) GBIF Occurrence Download

https://doi.org/10.15468/dl.3hyecd

Urticaceae

GBIF.org (23 March 2023) GBIF Occurrence Download

https://doi.org/10.15468/dl.u5aybs

Velloziaceae

GBIF.org (23 March 2023) GBIF Occurrence Download

https://doi.org/10.15468/dl.wk4qhy

Verbenaceae

GBIF.org (23 March 2023) GBIF Occurrence Download

https://doi.org/10.15468/dl.wschg7

Violaceae

GBIF.org (23 March 2023) GBIF Occurrence Download

https://doi.org/10.15468/dl.a9eh9v

Vitaceae

GBIF.org (23 March 2023) GBIF Occurrence Download

https://doi.org/10.15468/dl.aq4eft

Vivianiaceae

GBIF.org (23 March 2023) GBIF Occurrence Download

https://doi.org/10.15468/dl.bqnbbv

Vochysiaceae

GBIF.org (23 March 2023) GBIF Occurrence Download

https://doi.org/10.15468/dl.c7g9wn

Winteraceae

GBIF.org (23 March 2023) GBIF Occurrence Download

https://doi.org/10.15468/dl.t4grv6

Ximeniaceae

GBIF.org (23 March 2023) GBIF Occurrence Download

https://doi.org/10.15468/dl.yuwymb

Xyridaceae

GBIF.org (23 March 2023) GBIF Occurrence Download

https://doi.org/10.15468/dl.3ntb6j

Zingiberaceae

GBIF.org (23 March 2023) GBIF Occurrence Download

https://doi.org/10.15468/dl.sxnav9

Zygophyllaceae

GBIF.org (23 March 2023) GBIF Occurrence Download

https://doi.org/10.15468/dl.wc67ce
