# Supplementary material for: Protecting hidden treasures: Indigenous lands safeguard 50% of areas with the highest potential for angiosperm discoveries in Brazil—patterns and conservation priorities
Source: PLoS One. 2025 Jul 9;20(7):e0326507. doi: 10.1371/journal.pone.0326507 (PMC12240397; doi:10.1371/journal.pone.0326507)
Supplement: S3 Appendix — (PDF) [file pone.0326507.s003.pdf]

Protecting Hidden Treasures: Indigenous Lands Safeguard 50% of Areas with the Highest Potential for Angiosperm Discoveries in Brazil – Patterns and Conservation Priorities

Janaína Gomes-da-Silva[1,*]
Eimear Nic Lughadha[2]
Rafaela Campostrini Forzza[1,3]

[1]Jardim Botânico do Rio de Janeiro, Rua Pacheco Leão, 915, Rio de Janeiro, RJ, 2460–030,
Brazil.
[2]Science Directorate, Royal Botanic Gardens, Kew, Richmond, TW9 3AE, UK
[3]Instituto Chico Mendes de Conservação da Biodiversidade, Parque Nacional do Descobrimento, Bahia, Brazil.
[*] Author for Correspondence: jgomes_da_silva@yahoo.com.br

Supporting Information

APPENDIX S3. Details of the taxonomic effort questionnaire by interview.

NOME / NAME:

INSTITUIÇÃO E DEPARTAMENTO/ INSTITUTION AND DEPARTMENT:

CARGO / POSITION:

(1) Família botânica a qual contribui para a Flora e Funga do Brasil / Botanical family that contributes to the Flora and Funga of Brazil:

Por favor, marque com um "**x**" as questões abaixo/ Mark the following questions with an "X":

Baseado na premissa que o esforço taxonômico é um poderoso preditor do número de espécies descritas / Based on the premise that taxonomic effort is a powerful predictor of the number of described species

(2) Em sua área de especialização, o esforço taxonômico medido pelo número de taxonomistas; levando em consideração as últimas **quatro** décadas / Taxonomic effort measured by the number of taxonomists; taking into account the **last four decades**:

( ) aumentou/increased ( )diminuiu / decreased ( ) estabilizou / stable

(3) Em sua área de especialização, o esforço taxonômico medido pelo número de taxonomistas; levando em consideração exclusivamente a **última** década/ Taxonomic effort measured by the number of taxonomists; taking into account **the last decade**:

( ) aumentou/increased ( )diminuiu / decreased ( ) estabilizou / stable

Obrigada pela contribuição / Thanks for the contribution.

Janaina Gomes-da-Silva; Rafaela Forzza

Projeto: **INVESTIGANDO O DESCONHECIDO**
