# Supplementary material for: Protecting hidden treasures: Indigenous lands safeguard 50% of areas with the highest potential for angiosperm discoveries in Brazil—patterns and conservation priorities
Source: PLoS One. 2025 Jul 9;20(7):e0326507. doi: 10.1371/journal.pone.0326507 (PMC12240397; doi:10.1371/journal.pone.0326507)
Supplement: S4 Appendix — The species accumulation curve, and the fit of the nonlinear models to the data. According to Prediction Model Results: Gompertz 3P, Gompertz 4P, Logistic 3P, and Weibull Growth models for the top 10 angiosperm families with the highest potential for species discoveries. (PDF) [file pone.0326507.s004.pdf]

Protecting Hidden Treasures: Indigenous Lands Safeguard 50% of Areas with the Highest Potential for Angiosperm Discoveries in Brazil – Patterns and Conservation Priorities

Janaína Gomes-da-Silva[1,*]
Eimear Nic Lughadha[2]
Rafaela Campostrini Forzza[1,3]

[1]Jardim Botânico do Rio de Janeiro, Rua Pacheco Leão, 915, Rio de Janeiro, RJ, 2460–030, Brazil.
[2]Science Directorate, Royal Botanic Gardens, Kew, Richmond, TW9 3AE, UK
[3]Instituto Chico Mendes de Conservação da Biodiversidade, Parque Nacional do Descobrimento, Bahia, Brazil.
[*] Author for Correspondence: jgomes_da_silva@yahoo.com.br

Supporting Information

APPENDIX S4.

Temporal description of families of angiosperms species. The species accumulation curve, and the fit of the nonlinear models to the data. Gompertz 3P, Gompertz 4P, Logistic 3P, and Weibull Growth models for the top 10 angiosperm families with the highest potential for species discoveries.

Model
Gompertz 3P
Gompertz 4P
Logistic 3P
Weibull Growth

Araceae

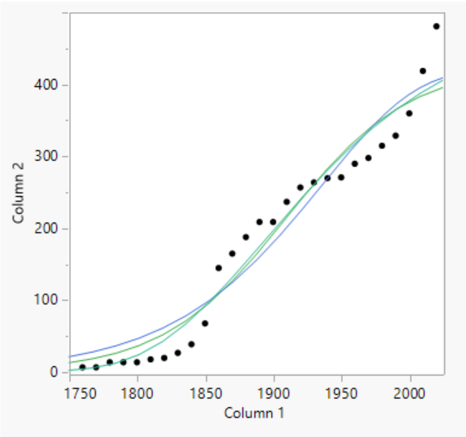

Apocynaceae

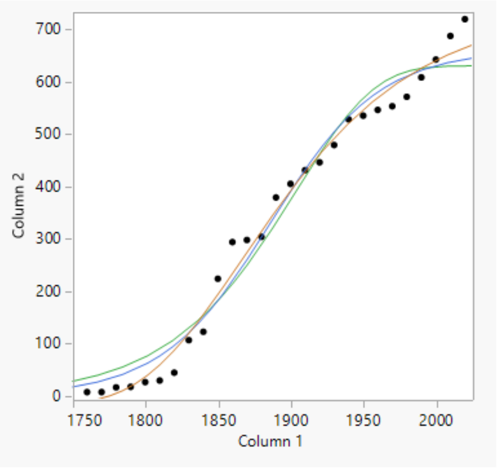

Asteraceae

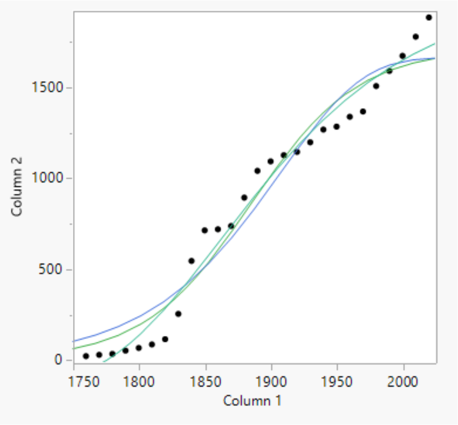

Bromeliaceae

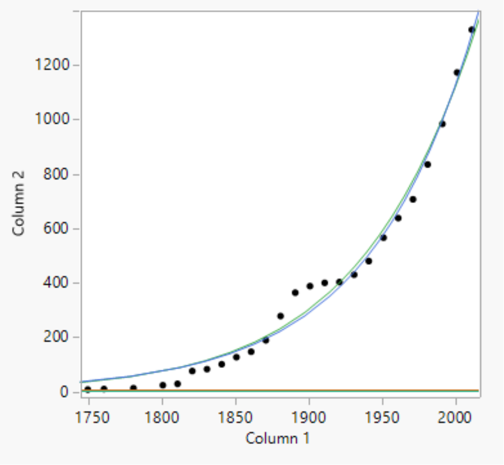

Cyperaceae

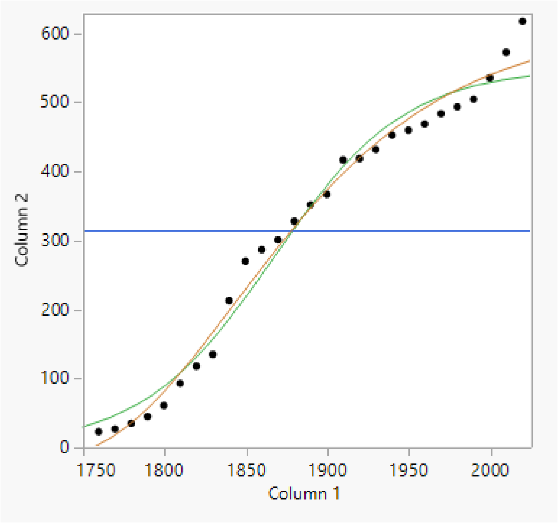

Euriocaulaceae

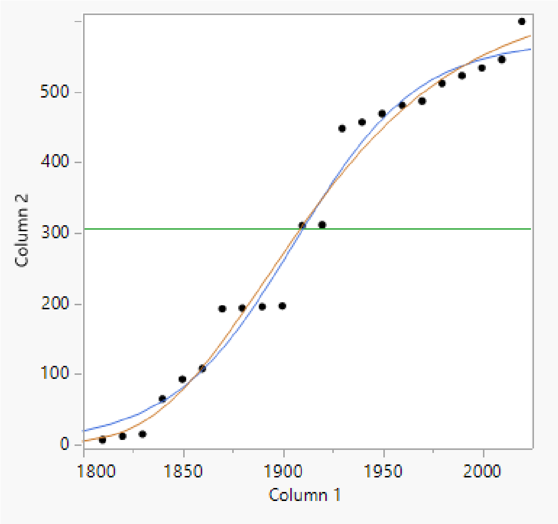

Euphorbiaceae

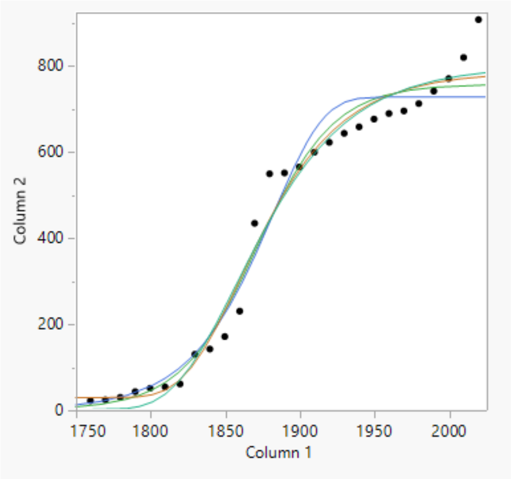

Fabaceae

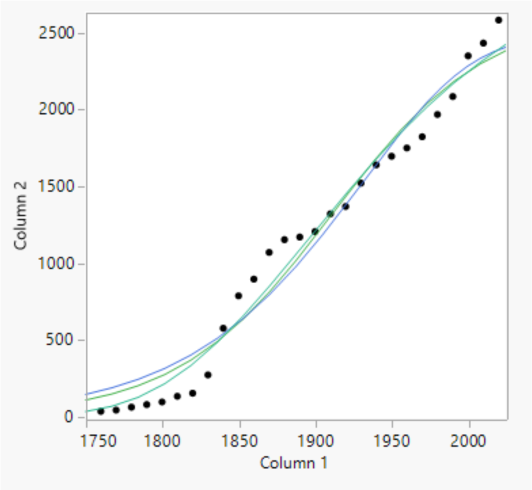

Lamiaceae

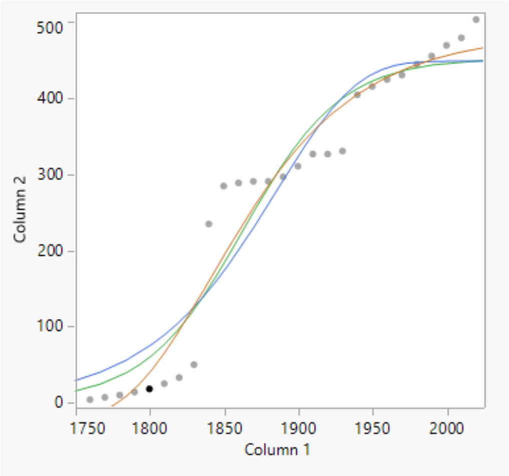

Malpighiaceae

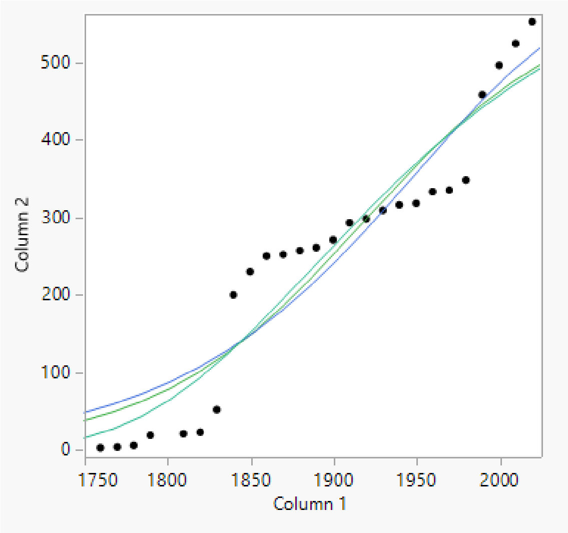

Malvaceae

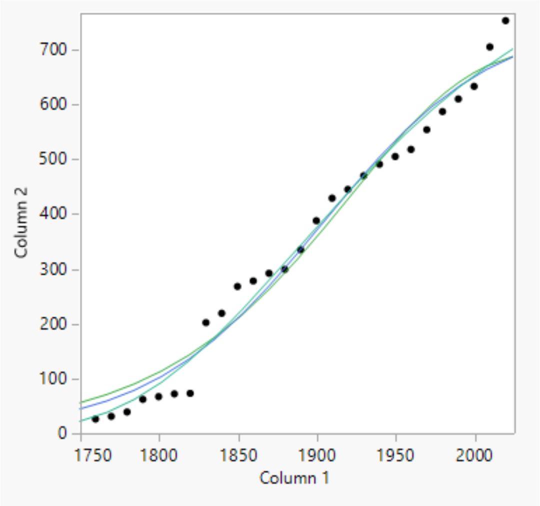

Melastomataceae

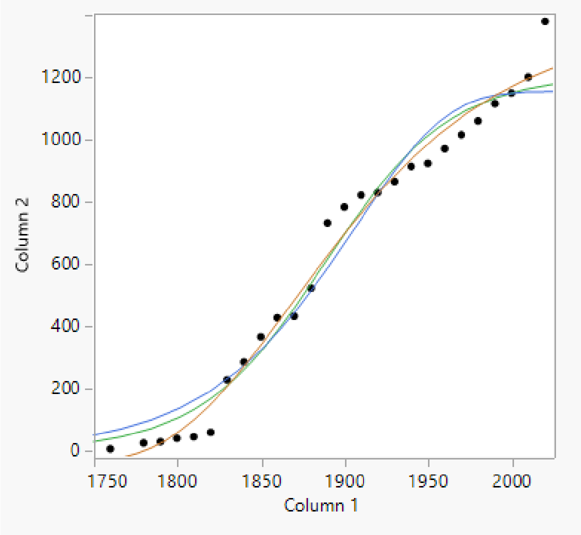

Myrtaceaae

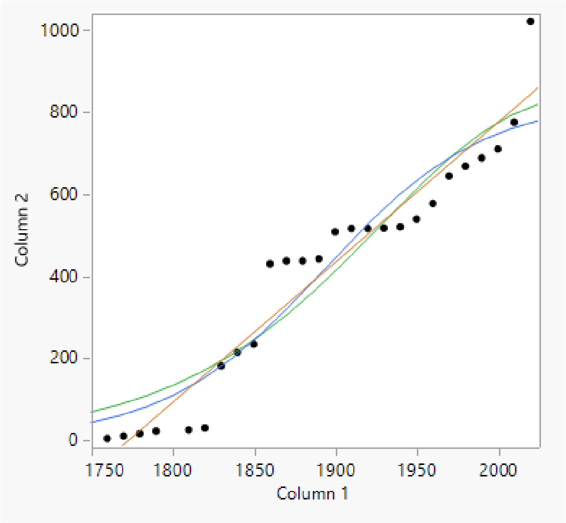

Orchidaceae

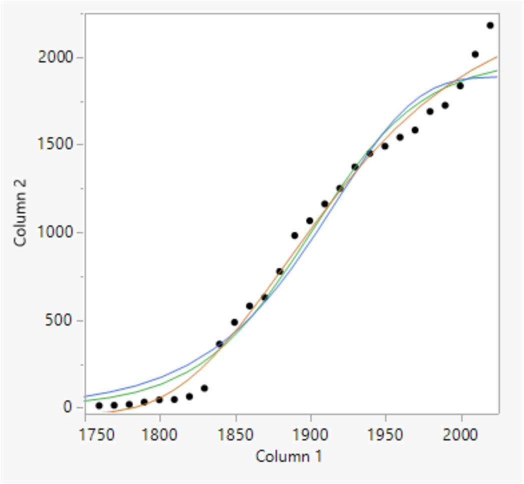

Poaceae

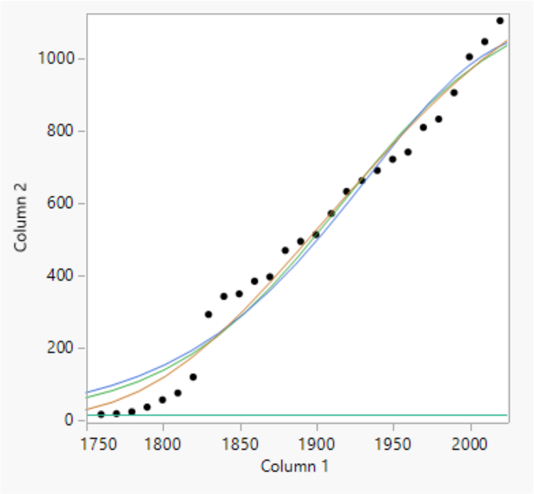

Rubiaceae

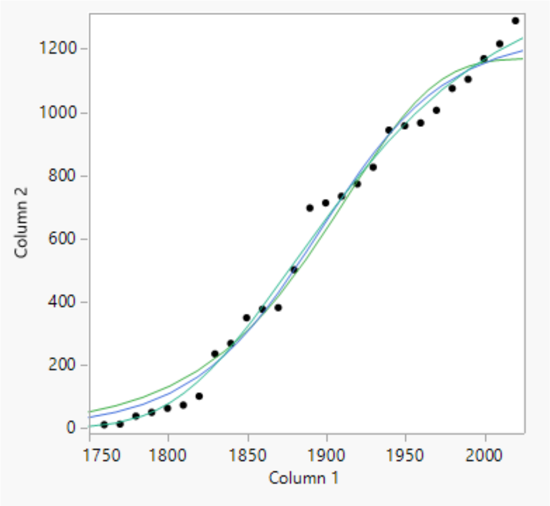

1200
1000
800
600
400
200
0
Column 2
1750
1800
1850
1900
1950
2000
Column 1
