# Supplementary material for: Protecting hidden treasures: Indigenous lands safeguard 50% of areas with the highest potential for angiosperm discoveries in Brazil—patterns and conservation priorities
Source: PLoS One. 2025 Jul 9;20(7):e0326507. doi: 10.1371/journal.pone.0326507 (PMC12240397; doi:10.1371/journal.pone.0326507)
Supplement: S5 Appendix — (PDF) [file pone.0326507.s005.pdf]

# Protecting Hidden Treasures: Indigenous Lands Safeguard 50% of Areas with the Highest Potential for Angiosperm Discoveries in Brazil – Patterns and Conservation Priorities

Janaína Gomes-da-Silva[1,*]
Eimear Nic Lughadha[2]
Rafaela Campostrini Forzza[1,3]

[1]Jardim Botânico do Rio de Janeiro, Rua Pacheco Leão, 915, Rio de Janeiro, RJ, 2460–030,
Brazil.
[2]Science Directorate, Royal Botanic Gardens, Kew, Richmond, TW9 3AE, UK
[3]Instituto Chico Mendes de Conservação da Biodiversidade, Parque Nacional do Descobrimento, Bahia, Brazil.
[*] Author for Correspondence: jgomes_da_silva@yahoo.com.br

## Supporting Information

**APPENDIX S5.** Table with parameter estimates and maximum-likelihood confidence intervals (CIs), Akaike's Information Criterion (AIC), AIC weight, and Bayesian Information Criterion (BIC) according to prediction model results: Gompertz 3P, Gompertz 4P, Logistic 3P, and Weibull Growth models for the top 10 angiosperm families with the highest potential for species discoveries.

| Fanily | Model | AICc | AICc Weight | BIC | Asymptote (species remainingto be described) |
|---|---|---|---|---|---|
| **Apocynaceae** | Gompertz 4P | 266,5126 | 1,00 | 270,1347 | 108 |
| **Araceae** | Gompertz 3P | 267,6397 | 0,97 | 271,0049 | 149 |
| **Asteraceae** | Gompertz 4P | 332,9871 | 0,99 | 336,6091 | 632 |
| **Bromeliaceae** | Gompertz 3P | 268 | 1,00 | 273 | 1058 |
| **Cyperaceae** | Gompertz 4P | 255,7436 | 1,00 | 259,3656 | 63 |
| **Eriocaulaceae** | Gompertz 4P | 220,4426 | 0,86 | 222,4539 | 126 |
| **Euphorbiaceae** | Gompertz 3P | 295,1903 | 0,59 | 298,5555 | 50 |
| **Fabaceae** | Gompertz 3P | 344,8856 | 1,00 | 348,2508 | 1193 |
| **Lamiaceae** | Gompertz 4P | 287,6614 | 0,92 | 291,2834 | 46 |
| **Malpighiaceae** | Gompertz 3P | 286,9348 | 0,85 | 290,0624 | 395 |
| **Malvaceae** | Gompertz 3P | 267,6613 | 0,99 | 271,0265 | 334 |
| **Melastomataceae** | Gompertz 4P | 296,0439 | 0,99 | 299,3344 | 196 |
| **Myrtaceae** | Gompertz 4P | 308,137 | 0,93 | 311,4275 | 651 |
| **Orchidaceae** | Gompertz 4P | 320,2314 | 1,00 | 323,8534 | 424 |
| **Poaceae** | Gompertz 3P | 295,6643 | 0,99 | 299,0295 | 703 |
| **Rubiaceae** | Gompertz 3P | 282,4368 | 1,00 | 285,8019 | 261 |
