# Supplementary material for: Protecting hidden treasures: Indigenous lands safeguard 50% of areas with the highest potential for angiosperm discoveries in Brazil—patterns and conservation priorities
Source: PLoS One. 2025 Jul 9;20(7):e0326507. doi: 10.1371/journal.pone.0326507 (PMC12240397; doi:10.1371/journal.pone.0326507)
Supplement: S6 Appendix — Comparison of models, number of known and predicted species, and percentage of expected increase, of flowering plants, by the Brazilian phytogeographic domains. For cases where the Weibull Growth model did not converge to real values (BIC number, AIC weight, or asymptote value), these are indicated by a ‘–’ in the table. (R) and (JMP) next to the model name indicate where the data was generated. Models without specifications indicate no differences in results between the execution programs. Models with the best fit for the evaluated database are highlighted in bold. (PDF) [file pone.0326507.s006.pdf]

Protecting Hidden Treasures: Indigenous Lands Safeguard 50% of Areas with the Highest Potential for Angiosperm Discoveries in Brazil – Patterns and Conservation Priorities

Janaína Gomes-da-Silva[1,*]
Eimear Nic Lughadha[2]
Rafaela Campostrini Forzza[1,3]

[1]Jardim Botânico do Rio de Janeiro, Rua Pacheco Leão, 915, Rio de Janeiro, RJ, 2460–030, Brazil.
[2]Science Directorate, Royal Botanic Gardens, Kew, Richmond, TW9 3AE, UK
[3]Instituto Chico Mendes de Conservação da Biodiversidade, Parque Nacional do Descobrimento, Bahia, Brazil.
[*] Author for Correspondence: jgomes_da_silva@yahoo.com.br

Supporting Information

APPENDIX S6. Complete table with all models and differences (when found) executed in R and JMP. For cases where the Weibull Growth model did not converge to real values (BIC number, AIC weight, or asymptote value), these are indicated by a '-' in the table. (R) and (JMP) next to the model name indicate where the data was generated. Models without specifications indicate no differences in results between the execution programs. Models with the best fit for the evaluated database are highlighted in bold.

Region	Model name	Current accumulation	Asymptote/ SR species remaining to be described	Lower 95%	Upper 95%	AIC (Akaike's Information Criterion)	AICc Weight	BIC(Bayesian Information Criterion)
**Amazon**	Gompertz 3P	11,903	1042	867	1216	405,1376	0,438005	408,50276
**Amazon**	Logistic 3P	11,903	805	545	1065	425,98275	1,30E-05	429,34792
**Amazon**	**Weibull Growth**	11,903	**1042**	**872**	**1211**	**404,63938**	**0,561908**	**408,00455**
**Amazon**	**Gompertz 3P ®**	11,903	**1403**	**748**	**2133**	**392,339**	**1**	**397,5223**
**Amazon**	Logistic 3P ®	11,903	488	-1325	469	432,9731	0	438,1564
**Amazon**	Weibull Growth ®	11,903	——	——	——	——	——	——
**Amazon**	**Gompertz 3P, basionym ®**	11,903	**3512**	**2571**	**4729**	**344**	**1**	**348,9795**
**Amazon**	Logistic 3P, basionym ®	11,903	817	224	1591	371,3059	0	376,4893
**Amazon**	Weibull Growth, basionym ®	11,903	——	——	——	——	——	——
**Amazon**	**Gompertz 3P, basionym (JMP)**	11,903	**3511**	**2503**	**4520**	**345,61432**	**0,999999**	**348,97948**
**Amazon**	Logistic 3P, basionym (JMP)	11,903	816	190	1443	373,12412	1,06E-06	376,48928
**Amazon**	Weibull Growth, basionym (JMP)	11,903	-128	-601	345	383,23655	6,77E-09	386,60172
**Amazon**	Taxonomic effort, all names	11,903	3205			563,8565	0,995504	567,744
**Amazon**	**Taxonomic effort, all names**	11,903	**1706**			**552,5408**	**0,993705**	**556,4283**
**Amazon**	Taxonomic effort, basionym	11,903	2762			270	0,5	274,3081
**Amazon**	**Taxonomic effort, basionym**	11,903	**4223**			**460**	**0,999948**	**463,9657**
**Amazon**	Average		**2566**	**2104**	**3087**			

**Atlantic Forest**	Gompertz 3P	14,905	1107	-1473	3687	448,74259	5,60E-05	452,10776
**Atlantic Forest**	Logistic 3P	14,905	1341	1050	1632	436,75184	0,022501	440,11701
**Atlantic Forest**	**Weibull Growth**	14,905	**1455**	**1189**	**1720**	**429,20966**	**0,977153**	**432,57482**
**Atlantic Forest**	**JMP Gompertz 3P, basionym**	14,905	**234**	**-1291**	**1759**	**417,47751**	**0,9985058**	**420,84268**
**Atlantic Forest**	Logistic 3P, basionym	14,905	1008	2049	33	430,52305	0,0014674	433,88822
**Atlantic Forest**	Weibull Growth, basionym	14,905	-1343	-2038	-380	438,52468	2,6854e-5	441,88985
**Atlantic Forest**	**Gompertz 3P, basionym®**	14,905	**234**	**-1151**	**2726**	**416**	**1**	**420,8427**
**Atlantic Forest**	Logistic 3P, basionym ®	14,905	1008	-1385	2054	428,7049	0	433,8882
**Atlantic Forest**	Weibull Growth, basionym ®	14,905	____	____	____	____	____	____
**Atlantic Forest**	Taxonomic effort, all names	14,905	**578**			**336,5724**	**1**	**340,4599**
**Atlantic Forest**	Taxonomic effort, all names	14,905	1172			566,6801	0,999598	570,5676
**Atlantic Forest**	Taxonomic effort, basionym	14,905	1925			479	0.6852662	482,7039
**Atlantic Forest**	Taxonomic effort, basionym	14,905	**3148**			**466,2374**	**0,994193**	**470,1249**
**Atlantic Forest**	Average		**1130**	**495**	**1986**			
**Caatinga**	Gompertz 3P	4,781	536	-73251	74322	404,66951	0,001909	408,03467
**Caatinga**	Logistic 3P	4,781	445	268	622	404,70649	0,001874	408,07165
**Caatinga**	**Weibull Growth**	4,781	**577**	**443**	**710**	**392,16992**	**0,988544**	**395,53508**

**Caatinga**	**Gompertz 3P, basionym**	4,781	**831**	**61**	**5166**	**346,1972**	**1**	**351,38053**
**Caatinga**	Logistic 3P, basionym	4,781	214	-357	2578	354,1448	0,018336	359,32814
**Caatinga**	Weibull Growth, basionym	4,781	____	____	____	____	____	____
**Caatinga**	**Taxonomic effort , all names**	4,781	**3640**			**314,0847**	**1**	**317,9722**
**Caatinga**	Taxonomic effort , all names	4,781	3191			525,2774	0,999911	529,1649
**Caatinga**	**Taxonomic effort, basionym**	4,781	**2825**			**396,515**	**0.9805329**	**400,4026**
**Caatinga**	Taxonomic effort, basionym	4,781	2122			205,3618	0,945499	209
**Caatinga**	Average		**1968**	**1742**	**3085**			
**Cerrado**	Gompertz 3P	12,025	579	463	696	384,73676	0,045054	388,10192
**Cerrado**	Logistic 3P	12,025	558	434	682	389,35234	0,004482	392,7175
**Cerrado**	**Weibull Growth**	12,025	**630**	**525**	**734**	**378,63863**	**0,950428**	**382,0038**
**Cerrado**	**Gompertz 3P, basionym**	12,025	**3**	**-1027**	**1021**	**403,01804**	**0,998217**	**406,38321**
**Cerrado**	Logistic 3P, basionym	12,025	____	____	____	____	____	____
**Cerrado**	Weibull Growth, basionym	12,025	____	____	____	____	____	____
**Cerrado**	Taxonomic effort, all names	12,025	524			541,348	1	545,2356
**Cerrado**	Taxonomic effort, all names	12,025	**675**			**540,2072**	**1**	**544,0947**
**Cerrado**	Taxonomic effort, basionym	12,025	605			370,0488	0,999016	373,9363
**Cerrado**	Taxonomic effort, basionym	12,025	**740**			**342,9918**	**0,991137**	**346,8793**
**Cerrado**	Average		**512**	**228**	**793**			
**Pampa**	Gompertz 3P	2,578	167	103	230	354,44433	0,069129	357,8095
**Pampa**	Logistic 3P	2,578	167	81	252	354,44433	0,069129	357,8095
**Pampa**	**Weibull Growth**	2,578	**209**	**135**	**283**	**350,18812**	**0,580614**	**353,55329**
**Pampa**	**Gompertz 3P, basionym**	2,578	**83**	**-159**	**158**	**310,32261**	**0,999559**	**313,68778**
**Pampa**	Logistic 3P, basionym	2,578	151	-216	229	325,7781	0,00044	329,14327
**Pampa**	Weibull Growth, basionym	2,578	195	-270	315	338,82804	6,46E-07	342,19321
**Pampa**	Taxonomic effort, all names	2,578	**596**			**235,847**	**1**	**239,7345**
**Pampa**	Taxonomic effort, all names	2,578	588			277,1137	1	281,0012

**Pampa**	Taxonomic effort, basionym	2,578	391			302,1668	1	306,0544
**Pampa**	Taxonomic effort, basionym	2,578	**115**			**282,1486**	**1**	**286,0361**
**Pampa**	Average		**251**	**171**	**288**			
**Pantanal**	Gompertz 3P	1470	135	94	176	326,97209	0,476238	330,33725
**Pantanal**	Logistic 3P	1470	106	-487	700	334,98534	0,008665	338,35051
**Pantanal**	**Weibull Growth**	1470	**135**	**95**	**175**	**326,95644**	**0,479978**	**330,32161**
**Pantanal**	**Gompertz 3P, basionym**	1470	**11**	**-26**	**59**	**219,3711**	**1**	**224,5544**
**Pantanal**	Logistic 3P, basionym	1470	43	36	61	233,4065	0,000895	238,58982
**Pantanal**	Weibull Growth, basionym	1470	65	-87	105	250,1418	2,08E-07	255,3251
**Pantanal**	Taxonomic effort, all names	1470	251			327,1492	0.9999997	331,0367
**Pantanal**	**Taxonomic effort, all names**	1470	**116**			**320,8431**	**0,999996**	**324,7307**
**Pantanal**	Taxonomic effort, basionym	1470	255			146,9947	1	150,8822
**Pantanal**	**Taxonomic effort, basionym**	1470	**157**			**135,8462**	**0,999998**	**139,7337**
**Pantanal**	Average		**105**	**86**	**127**			
**Total dominions**	**W. G., G. 3P, all names**		**4409**		**5755**			
**Total dominions**	**W. G., G. 3P, basionym**		**4674**		**12892**			
**Total dominions**	**Taxonomic effort , all names**		**7311**					
**Total dominions**	**Taxonomic effort, basionym**		**11208**					
**Angiosperms**	Taxonomic effort, all names		11749			665,6512	0.927482	669,5387
**Angiosperms**	Taxonomic effort, all names		**12862**			**664,1647**	**1**	**668,0522**
**Angiosperms**	Taxonomic effort, basionym		**10107**			**652,7798**	**1**	**656,6673**
**Angiosperms**	Taxonomic effort, basionym		6823			645,3386	0,966498	649,2261
