# Supplementary material for: Protecting hidden treasures: Indigenous lands safeguard 50% of areas with the highest potential for angiosperm discoveries in Brazil—patterns and conservation priorities
Source: PLoS One. 2025 Jul 9;20(7):e0326507. doi: 10.1371/journal.pone.0326507 (PMC12240397; doi:10.1371/journal.pone.0326507)
Supplement: S7 Appendix — (PDF) [file pone.0326507.s007.pdf]

# Protecting Hidden Treasures: Indigenous Lands Safeguard 50% of Areas with the Highest Potential for Angiosperm Discoveries in Brazil – Patterns and Conservation Priorities

Janaína Gomes-da-Silva[1,*]
Eimear Nic Lughadha[2]
Rafaela Campostrini Forzza[1,3]

[1]Jardim Botânico do Rio de Janeiro, Rua Pacheco Leão, 915, Rio de Janeiro, RJ, 2460–030, Brazil.
[2]Science Directorate, Royal Botanic Gardens, Kew, Richmond, TW9 3AE, UK
[3]Instituto Chico Mendes de Conservação da Biodiversidade, Parque Nacional do Descobrimento, Bahia, Brazil.
[*] Author for Correspondence: jgomes_da_silva@yahoo.com.br

## Supporting Information

**Appendix S7**. Cumulative historical deforestation data up to 2022, retrieved from the Amazon Deforestation Estimation Project (PRODES), was used to assess its correlation with collection efforts in the region up to 2020.

| no/Estados | AC | AM | AP | MA | MT | PA | RO | RR | TO | AMZ LEGAL |
|---|---|---|---|---|---|---|---|---|---|---|
| **1988** | 620 | 1510 | 60 | 2450 | 5140 | 6990 | 2340 | 290 | 1650 | 21050 |
| **1989** | 540 | 1180 | 130 | 1420 | 5960 | 5750 | 1430 | 630 | 730 | 17770 |
| **1990** | 550 | 520 | 250 | 1100 | 4020 | 4890 | 1670 | 150 | 580 | 13730 |
| **1991** | 380 | 980 | 410 | 670 | 2840 | 3780 | 1110 | 420 | 440 | 11030 |
| **1992** | 400 | 799 | 36 | 1135 | 4674 | 3787 | 2265 | 281 | 409 | 13786 |
| **1993** | 482 | 370 | 0 | 372 | 6220 | 4284 | 2595 | 240 | 333 | 14896 |
| **1994** | 482 | 370 | 0 | 372 | 6220 | 4284 | 2595 | 240 | 333 | 14896 |

| | | | | | | | | | | |
|---|---|---|---|---|---|---|---|---|---|---|
| **1995** | 1208 | 2114 | 9 | 1745 | 10391 | 7845 | 4730 | 220 | 797 | 29059 |
| **1996** | 433 | 1023 | 0 | 1061 | 6543 | 6135 | 2432 | 214 | 320 | 18161 |
| **1997** | 358 | 589 | 18 | 409 | 5271 | 4139 | 1986 | 184 | 273 | 13227 |
| **1998** | 536 | 670 | 30 | 1012 | 6466 | 5829 | 2041 | 223 | 576 | 17383 |
| **1999** | 441 | 720 | 0 | 1230 | 6963 | 5111 | 2358 | 220 | 216 | 17259 |
| **2000** | 547 | 612 | 0 | 1065 | 6369 | 6671 | 2465 | 253 | 244 | 18226 |
| **2001** | 419 | 634 | 7 | 958 | 7703 | 5237 | 2673 | 345 | 189 | 18165 |
| **2002** | 883 | 885 | 0 | 1085 | 7892 | 7510 | 3099 | 84 | 212 | 21651 |
| **2003** | 1078 | 1558 | 25 | 993 | 10405 | 7145 | 3597 | 439 | 156 | 25396 |
| **2004** | 728 | 1232 | 46 | 755 | 11814 | 8870 | 3858 | 311 | 158 | 27772 |
| **2005** | 592 | 775 | 33 | 922 | 7145 | 5899 | 3244 | 133 | 271 | 19014 |
| **2006** | 398 | 788 | 30 | 674 | 4333 | 5659 | 2049 | 231 | 124 | 14286 |
| **2007** | 184 | 610 | 39 | 631 | 2678 | 5526 | 1611 | 309 | 63 | 11651 |
| **2008** | 254 | 604 | 100 | 1271 | 3258 | 5607 | 1136 | 574 | 107 | 12911 |
| **2009** | 167 | 405 | 70 | 828 | 1049 | 4281 | 482 | 121 | 61 | 7464 |
| **2010** | 259 | 595 | 53 | 712 | 871 | 3770 | 435 | 256 | 49 | 7000 |
| **2011** | 280 | 502 | 66 | 396 | 1120 | 3008 | 865 | 141 | 40 | 6418 |
| **2012** | 305 | 523 | 27 | 269 | 757 | 1741 | 773 | 124 | 52 | 4571 |
| **2013** | 221 | 583 | 23 | 403 | 1139 | 2346 | 932 | 170 | 74 | 5891 |
| **2014** | 309 | 500 | 31 | 257 | 1075 | 1887 | 684 | 219 | 50 | 5012 |
| **2015** | 264 | 712 | 25 | 209 | 1601 | 2153 | 1030 | 156 | 57 | 6207 |
| **2016** | 372 | 1129 | 17 | 258 | 1489 | 2992 | 1376 | 202 | 58 | 7893 |
| **2017** | 257 | 1001 | 24 | 265 | 1561 | 2433 | 1243 | 132 | 31 | 6947 |
| **2018** | 444 | 1045 | 24 | 253 | 1490 | 2744 | 1316 | 195 | 25 | 7536 |
| **2019** | 682 | 1434 | 32 | 237 | 1702 | 4172 | 1257 | 590 | 23 | **10129** |
| **2020** | 706 | 1512 | 24 | 336 | 1779 | 4899 | 1273 | 297 | 25 | **10851** |

| | | | | | | | | | | |
|---|---|---|---|---|---|---|---|---|---|---|
| **2021** | 889 | 2306 | 17 | 350 | 2213 | 5238 | 1673 | 315 | 37 | **13038** |
| **2022*** | 847 | 2607 | 6 | 282 | 1906 | 4141 | 1512 | 240 | 27 | **11568** |
| | | | | | | | | | | **481844** |
| **2022-2021*** | **-5%** | **13%** | **-65%** | **-19%** | **-14%** | **-21%** | **-10%** | **-24%** | **-27%** | |

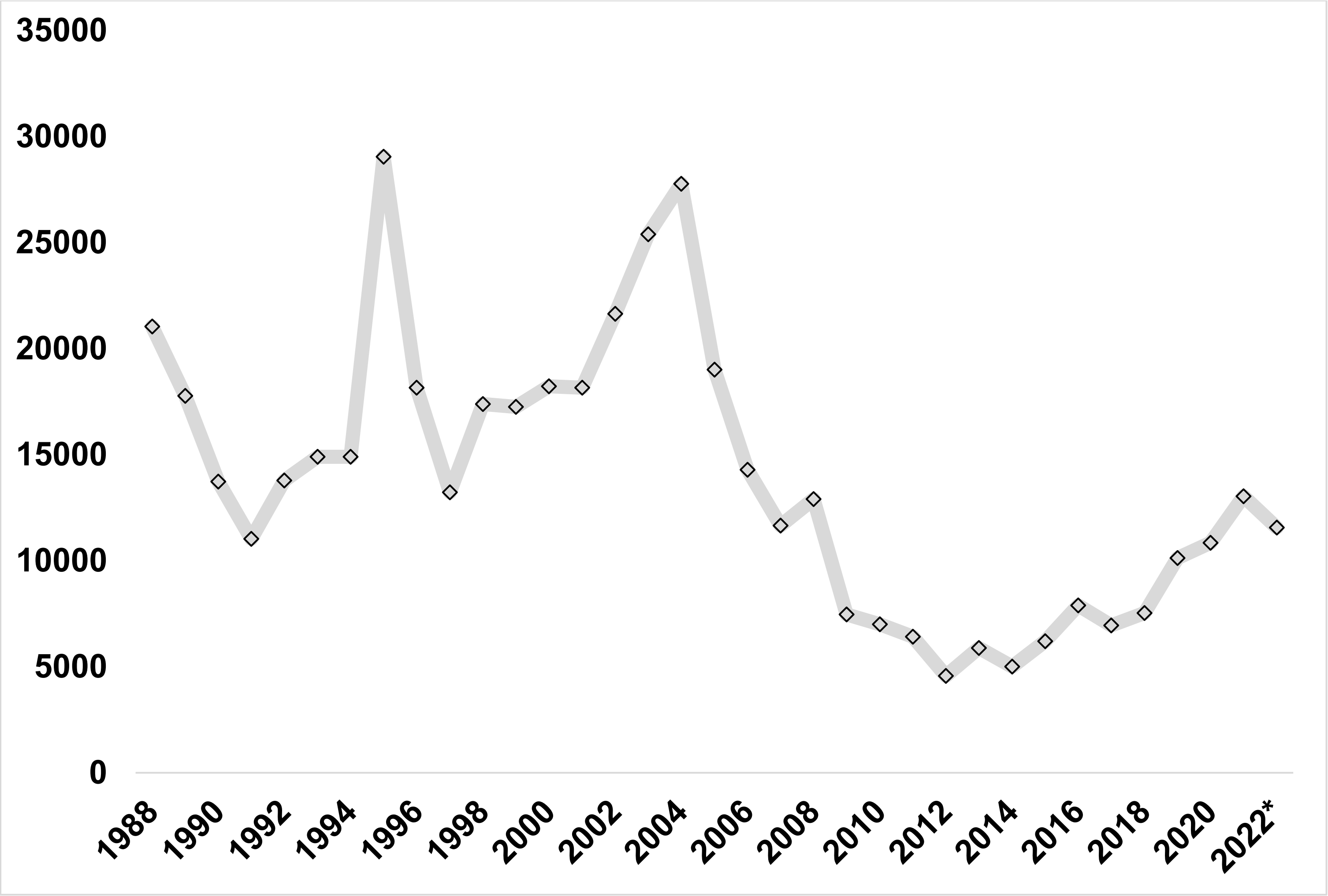
35000
30000
25000
20000
15000
10000
5000
0
1988
1990
1992
1994
1996
1998
2000
2002
2004
2006
2008
2010
2012
2014
2016
2018
2020
2022*
