# Supplementary material for: Protecting hidden treasures: Indigenous lands safeguard 50% of areas with the highest potential for angiosperm discoveries in Brazil—patterns and conservation priorities
Source: PLoS One. 2025 Jul 9;20(7):e0326507. doi: 10.1371/journal.pone.0326507 (PMC12240397; doi:10.1371/journal.pone.0326507)
Supplement: S1 Fig — (PDF) [file pone.0326507.s008.pdf]

Protecting Hidden Treasures: Indigenous Lands Safeguard 50% of Areas with the Highest Potential for Angiosperm Discoveries in Brazil – Patterns and Conservation Priorities

Janaína Gomes-da-Silva[1,*]
Eimear Nic Lughadha[2]
Rafaela Campostrini Forzza[1,3]

[1]Jardim Botânico do Rio de Janeiro, Rua Pacheco Leão, 915, Rio de Janeiro, RJ, 2460–030, Brazil.
[2]Science Directorate, Royal Botanic Gardens, Kew, Richmond, TW9 3AE, UK
[3]Instituto Chico Mendes de Conservação da Biodiversidade, Parque Nacional do Descobrimento, Bahia, Brazil.
[*] Author for Correspondence: jgomes_da_silva@yahoo.com.br

Supporting Information

Fig. S1. Completeness of the taxonomic inventory by polygon at 1/10° and ½° resolutions, with yellow circles highlighting areas with the broadest gaps and lowest completeness (a-f). Density analysis, geohash multi-layer density map (g) and a comprehensive map (h) show regions with the highest potential for future angiosperm discoveries, superimposed on the Brazilian phytogeographic domains. White areas indicate regions that emerge as holding the greatest discovery opportunities, and dark green indicate the lowest opportunity.

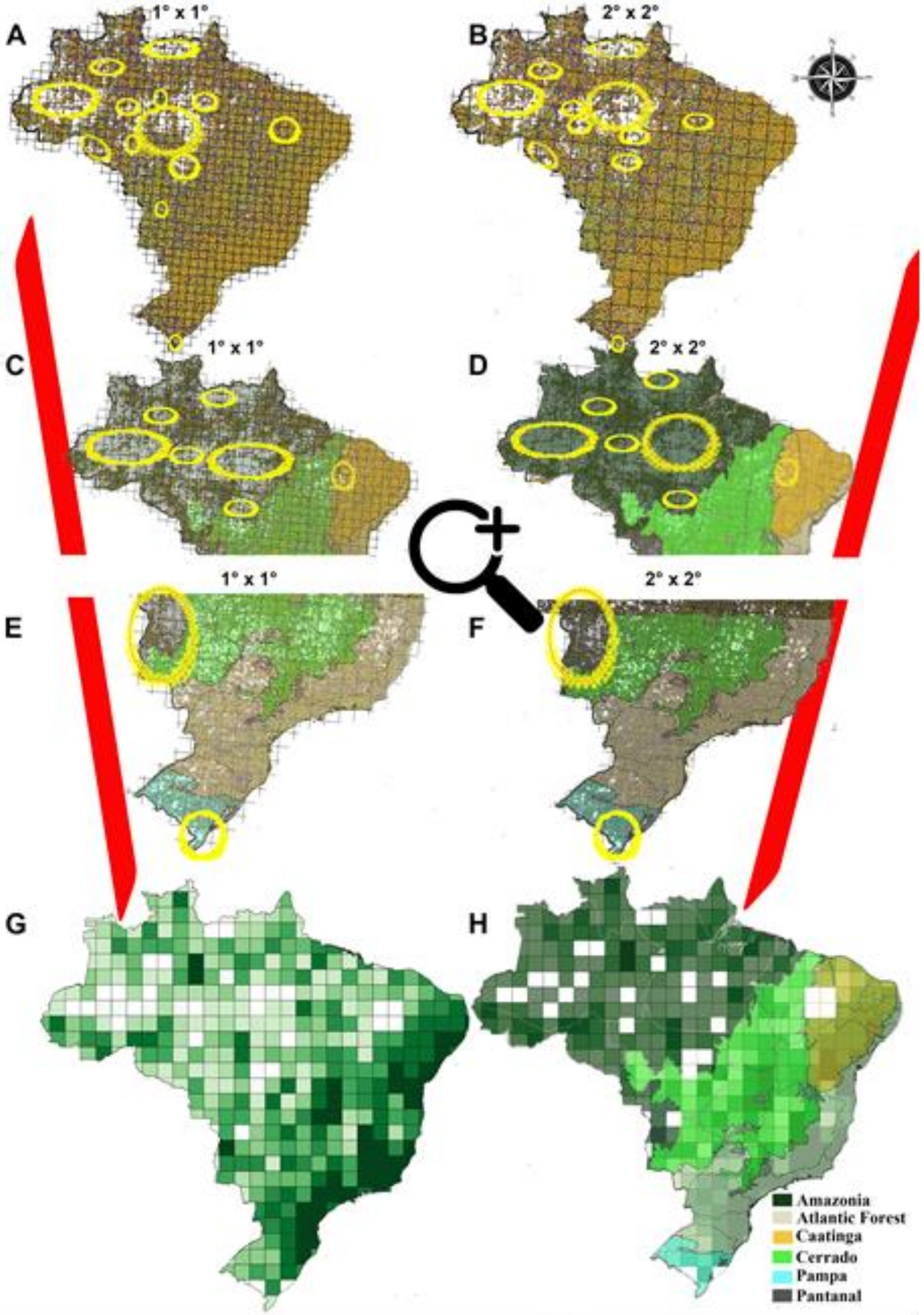

A
1° x 1°
B
2° x 2°
C
1° x 1°
D
2° x 2°
E
1° x 1°
F
2° x 2°
G
H
Amazonia
Atlantic Forest
Caatinga
Cerrado
Pampa
Pantanal
